# PyMiner: A method for metabolic pathway design based on the uniform similarity of substrate-product pairs and conditional search

**DOI:** 10.1371/journal.pone.0266783

**Published:** 2022-04-11

**Authors:** Xinfang Song, Mingyu Dong, Min Liu

**Affiliations:** Department of Automation, Tsinghua University, Beijing, China; Chinese Academy of Sciences, CHINA

## Abstract

Metabolic pathway design is an essential step in the course of constructing an efficient microbial cell factory to produce high value-added chemicals. Meanwhile, the computational design of biologically meaningful metabolic pathways has been attracting much attention to produce natural and non-natural products. However, there has been a lack of effective methods to perform metabolic network reduction automatically. In addition, comprehensive evaluation indexes for metabolic pathway are still relatively scarce. Here, we define a novel uniform similarity to calculate the main substrate-product pairs of known biochemical reactions, and develop further an efficient metabolic pathway design tool named PyMiner. As a result, the redundant information of general metabolic network (GMN) is eliminated, and the number of substrate-product pairs is shown to decrease by 81.62% on average. Considering that the nodes in the extracted metabolic network (EMN) constructed in this work is large in scale but imbalanced in distribution, we establish a conditional search strategy (CSS) that cuts search time in 90.6% cases. Compared with state-of-the-art methods, PyMiner shows obvious advantages and demonstrates equivalent or better performance on 95% cases of experimentally verified pathways. Consequently, PyMiner is a practical and effective tool for metabolic pathway design.

## Introduction

With the rapid development of metabolic engineering and synthetic biology, an increasing number of engineered microorganisms have been successfully developed to produce various natural and non-natural chemicals, such as isobutanol, artemisinin and so forth [1]. And metabolic pathway design is the first and possibly the most critical step to develop a high-yielding microbial strain for the production of high value-added chemicals. Currently, metabolic pathway design methods can be roughly divided into two categories: the first type of methods utilizes existing biochemical reactions to construct biosynthesis pathways toward target products, such as FMM [2] and MRE [3]; the second type of methods employs the promiscuity of enzymes to design de novo biochemical reactions, which are represented by BNICE [4], RetroPath [5] and THERESA [6]. The main difference between the above two types of methods is whether exploiting de novo biochemical reactions to construct metabolic pathways. The latter expands the biochemical reaction space through reaction rules. However, due to the high complexity of in silico enzyme design, the former is the most direct, and usually the most commonly applied, metabolic pathway design method [7].

Table 1 lists some representative methods for metabolic pathway design of the former category and summarizes their characteristics from various perspectives. These approaches first search possible pathways by mining the known biochemical reaction space, and then rank candidate pathways based on the metabolic network of one specific chassis microorganism (except NICEpath [8]). However, most of the reported methods only employ biochemical reactions from a single database (except EcoSynther [9]), and this may limit the search space due to the lack of complementary information from different databases. In addition, there is so far no comprehensive evaluation that simultaneously considers the following indexes: the metabolic burden of chassis strains, the atom utilization of initial substrate, the atom conservation of target product, and the maximum synthesis rate of target product.

**Table 1:** Summary of metabolic pathway design methods and corresponding characteristics.

Method	Database	Chassis	Network pruning	Search algorithm	Pathway ranking	Interface	(Ref.)
PyMiner	KEGG, Rhea, MetaCyc	Multiple choices	Manual cofactor removal and atom mapping	BFS and DFS based on LTIOD	Infeasible/foreign/native length, atom utilization/conservation, and main metabolic flux	Python	This study
PHT	KEGG	Multiple choices	—	BFS with HOHL	Pathway length and structure similarity	Web-based	[10]
MetaRoute	KEGG	Multiple choices	Atom mapping and weighted graph	Eppstein’s k-shortest path	Compound connectivity and atom conservation	Web-based	[11]
FMM	KEGG	Multiple choices	Manual cofactor removal	BFS	—	Web-based	[2]
RouteSearch	MetaCyc	Multiple choices	Atom mapping	Branch and Bound	Pathway length and atom conservation	Web-based	[14]
MRE	KEGG	Multiple choices	Weighted graph	Yen’s k-shortest path	Thermodynamics and competitive reaction	Web-based	[3]
EcoSynther	KEGG, Rhea	*Escherichia coli*	—	Probabilistic-based algorithm	Pathway length	Web-based	[9]
PATH^cre8^	KEGG	Multiple choices	Weighted graph	Yen’s k-shortest path	Pathway score	Web-based	[12]
NICEpath	KEGG	—	Weighted graph	Yen’s k-shortest path	Pathway score	Python	[8]

The abbreviations are: BFS, breadth-first search; DFS, depth-first search; HOHL, higher-order horn logic and LTIOD, local total in-out degree.

The efficiency of pathway design method can be improved by constructing substrate-product pairs of all available biochemical reactions and further establishing a reduced metabolic network, as previously studied by PHT [10], MetaRoute [11], MRE, PATH^cre8^ [12] and NICEpath. In these methods, PHT takes the product of molecular structure similarity and atomic mass contribution ratio as the basis to construct substrate-product pairs, and then employs single-step structure similarity and global source-target structure similarity to narrow the search space. MetaRoute adopts atom mapping rules to all reactions and constructs substrate-product pairs with atom transfer. NICEpath applies conserved atom ratio to construct weighted substrate-product pairs of all reactions. This method counts all the non-hydrogen atoms in the same substrate or product, but fails to reflect the influence of stoichiometric coefficient. However, high atom conservation in single step does not guarantee equivalent conservation in complete metabolic pathway. MRE directly utilizes RPAIR from KEGG [13] to restrict the search space of metabolic pathway. Furthermore, an efficient and automatic approach for the redundant information reduction of metabolic network still remains a challenge.

Biologically infeasible metabolic pathways can be avoided, as demonstrated by MetaRoute, RouteSearch [14] and AGPathFinder [15], via tracing the atom transfer route in metabolic pathways. MetaRoute traces the transfer route of substrate atoms by sequentially adopting atom mapping rules to each extracted path, and then excludes pathways without atoms transferred to target product. In contrast, RouteSearch applies atom tracing to the process of metabolic pathway search, that is, it uses weighted index, consisting of the number of atoms lost from initial substrate, the length of endogenous steps, and the length of exogenous steps, to guide the search process. Moreover, AGPathFinder introduces atomic group tracking to guide the process of pathway inference.

**Fig 1:**
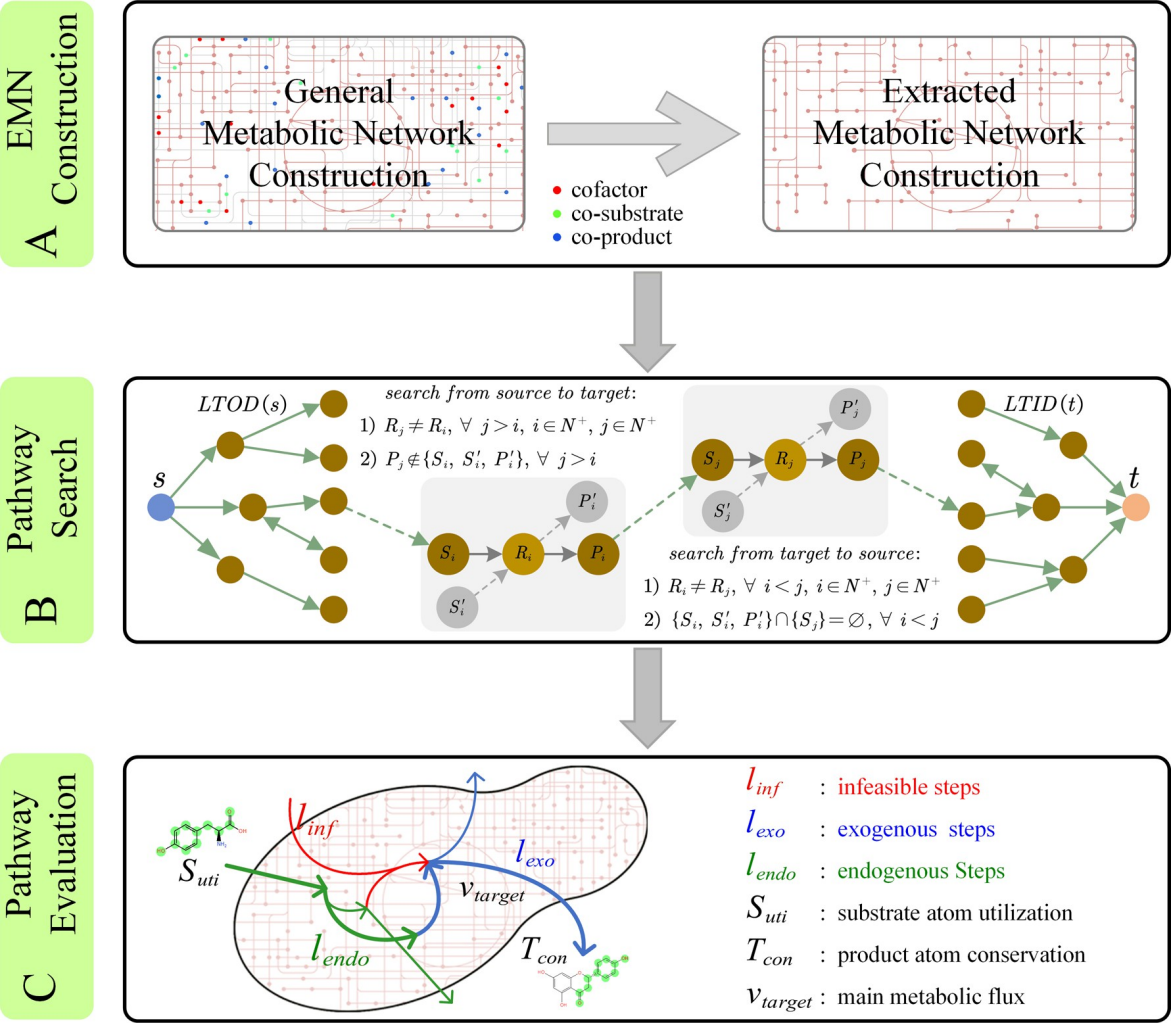
The workflow of PyMiner. (A) The construction of extracted metabolic network (EMN). The uniform similarity of substrate-product pairs was employed to construct EMN. (B) Metabolic pathway search. A conditional search strategy (CSS) established in this work was applied to cut search time. The green dashed arrow means multi-step reactions and the gray dashed arrow indicates implicit access of compound nodes (substrates or products). (C) Metabolic pathway evaluation. An exquisite set of evaluation indexes were adopted to evaluate and rank all the extracted metabolic pathways. See ‘Materials and methods’ section for more details.

In this paper, we develop an effective approach for metabolic pathway design called PyMiner based on the uniform similarity of substrate-product pairs. PyMiner mainly consists of three parts (Fig 1): 1) extracted metabolic network (EMN) construction, 2) metabolic pathway search, and 3) metabolic pathway evaluation. We summarize our key novelty and contribution in three aspects. Firstly, based on the uniform similarity, redundant information contained in general metabolic network (GMN) is removed, leading to an average decrease level of 81.62% of the number of substrate-product pairs. Secondly, a conditional search strategy (CSS) based on local total in-out degree (LTIOD) effectively cuts the time cost in 90.6% cases and further enhances the search efficiency. Finally, by tracing atom transfer route, PyMiner excludes infeasible metabolic pathways without atoms (such as carbon atom) transferring from the initial substrate to the target product, and then grants priority to pathways with both higher initial substrate atom utilization and higher target product atom conservation. Compared with recently reported methods for metabolic pathway design, PyMiner has obvious advantages, and performs equally or better on 95% cases of experimentally verified pathways. Furthermore, in terms of presenting complete details and predicting optimization space of the selected metabolic pathway, PyMiner offers definite improvement and valuable information. In brief, PyMiner is a practical and effective method for metabolic pathway design, which can extensively mine the existing biochemical space and retrieve biologically feasible metabolic pathways.

## Materials and methods

### Data resources

To make full use of the existing knowledge in various biochemical databases, we extracted sufficient data including compounds and biochemical reactions from KEGG, ChEBI [16], Rhea [17] and MetaCyc [18]. However, the data derived from these databases suffers from some key information missing, conflicting, and so forth, which cannot be directly used for pathway search before data pre-treatment. Firstly, considering that the atom mapping information of all biochemical reactions needs to be computed in next stage, we reconciled the structure information of some compounds (e.g. acceptor) based on the reference relationships among above-mentioned databases in the data cleaning process. Then we abandoned some biochemical reactions of which the structure information of corresponding substrates or products were still missing. Secondly, in order to reduce the information redundancy, we constructed a new database called KndPad (**kn**owledge **d**atabase of **pa**thway **d**esign) by fusing the obtained data, and unified the structure information of compounds in KEGG, ChEBI and Rhea with the reference of compounds in MetaCyc. Meanwhile, we took the direction of the biochemical reactions from MetaCyc as a reference to unify the reaction direction from KEGG and Rhea, and then classified other reactions with uncertain direction as reversible ones. 144,175 compounds and 27,655 reactions were conclusively integrated into KndPad database.

**Fig 2:**
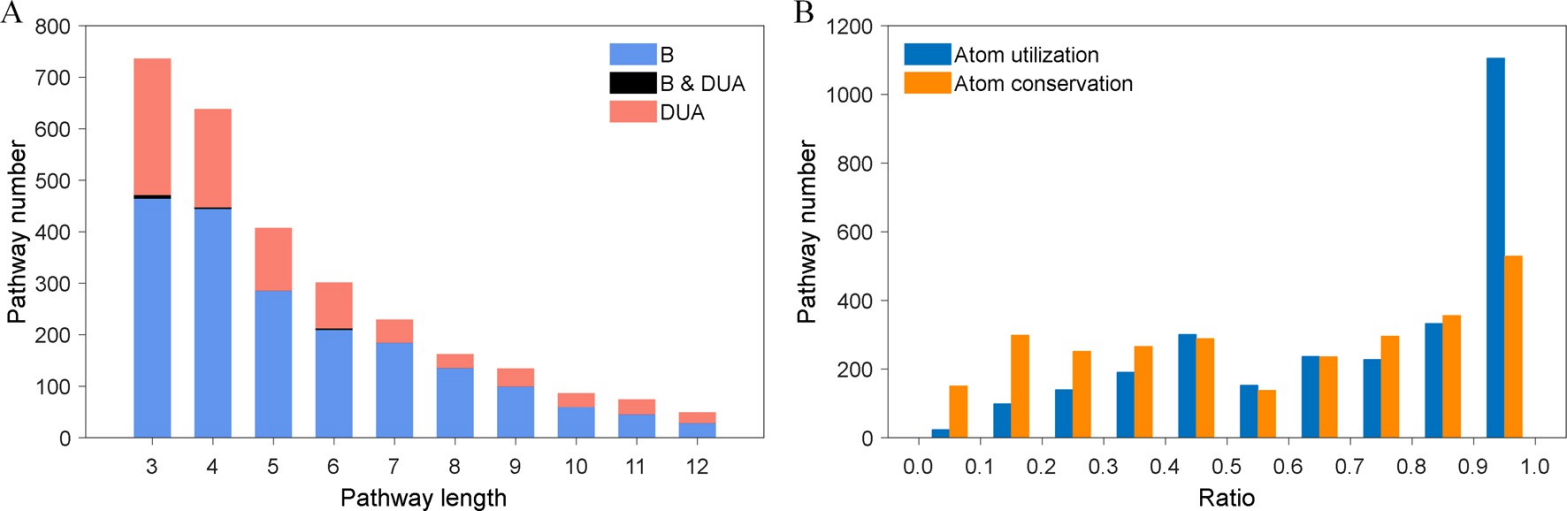
The statistical information of the validation dataset on metabolic pathway. (A) The length distribution of metabolic pathways. B represents the superclass of Biosynthesis, and DUA denotes the superclass of Degradation/Utilization/Assimilation. (B) The utilization distribution of non-hydrogen and critical atoms of the initial substrates (blue columns), and the conservation distribution of non-hydrogen and critical atoms of the target products (orange columns).

Finally, in order to verify the effectiveness of the pathway design method, we constructed a validation dataset containing 2812 linear and finite-length metabolic pathways from the Biosynthesis superclass and the Degradation/Utilization/Assimilation superclass in MetaCyc. As a result, a variety of metabolic pathways are contained, such as amino acid biosynthesis, secondary metabolite biosynthesis and alcohol degradation. The statistical information of this validation dataset is shown in Fig 2, which is in accord with the requirement of most pathway design. The length of these metabolic pathways is distributed between 3 and 12 (Fig 2A), while 91% of the metabolic pathways have a substrate-atom utilization greater than 0.3, and 75% have a product-atom conservation greater than 0.3 (Fig 2B). The statistical information of compounds, biochemical reactions and metabolic pathways from the integrated database KndPad and the original databases that adopted in this paper are summarized in Table 2. Eventually, the cleaned data of KndPad and the detailed validation data are provided in S1 Table.

**Table 2:** Statistical information of compounds, reactions and metabolic pathways in major biochemical databases.

Database	Compounds	Reactions	Pathways
KndPad _(Version 1.0)_	144,175	27,655	2,812
KEGG _(Version 97.0)_	18,776	11,496	—
ChEBI _(Version 196)_	134,146	—	—
Rhea _(Version 117)_	10,861	13,353	—
MetaCyc _(Version 24.5)_	21,450	18,520	3,241*

* The number of complicated and nonlinear metabolic pathways in MetaCyc.

### Constructing the extracted metabolic network

Utilizing the obtained reaction and compound data, bipartite graph could be applied to construct the GMN for pathway search. In this traditional method, nodes represent the compounds and reactions, edges represent the corresponding relationship, and the direction of each edge can also be clearly illustrated [12]. However, there still is a large amount of redundant information retained in GMN, even for KndPad. As a consequence, directly retrieving metabolic pathways in GMN will not only increase computational costs, but also return considerable biologically meaningless results. To address this problem, we have automatically calculated substrate-product pairs to simplify GMN and further established an EMN. The atom mapping tool RDT [19] was applied to generate atom mapping information. In addition, the pseudo codes of EMN construction are provided in S2 File.

#### Construction of general cofactors.

Generally, the efficiency and the quality of pathway search can be improved by constructing general cofactors (GC) and excluding ineligible metabolites during the initialization of metabolic network [8, 12]. In order to keep the diversity of biochemical reactions as much as possible, the GC set (see details in S2 Table) can be divided into three subsets instead of one that utilized in the previous studies [8,12]: single family (such as H_2_O, CoA and K^+^), double family (such as NADP^+^/NADPH and NAD^+^/NADH) and quadruple family (such as ATP/ADP/AMP/Adenosine and UTP/UDP/UMP/Uridine). Different strategies are adopted to deal with these cofactors, including direct exclusion of the cofactors belonging to single family, and conditional exclusion of the cofactors belonging to the double family or quadruple family that located on both sides of one reaction with an identical coefficient.

#### Definition of uniform similarity.

In metabolic pathway analysis, we are more concerned about the atoms transferred from initial substrate to target product. Hence instead of molecular fingerprints [10], atom transfer information is utilized to define a similarity between substrates and products. Here, we define a uniform similarity to evaluate the similarity between substrates and products in biochemical reactions. Without loss of generality, one biochemical reaction *R*_*X*_ is written as:

$$\lambda_{1}S_{1}+\lambda_{2}S_{2}+\cdots +\lambda_{i}S_{i}+\cdots ⟷\mu_{1}P_{1}+\mu_{2}P_{2}+\cdots +\mu_{j}P_{j}+\cdots$$ (1)

where, *S*_*i*_ and *P*_*j*_ denote the substrate and product respectively, *λ*_*i*_∈*N*^+^ and *μ*_*j*_∈*N*^+^ indicate their coefficients. The Eq (1) can be expanded as follows:

$$\sum_{k=1}^{\lambda_{1}}S_{1}^{k}+\sum_{k=1}^{\lambda_{2}}S_{2}^{k}+\cdots +\sum_{k=1}^{\lambda_{i}}S_{i}^{k}+\cdots ⟷\sum_{l=1}^{\mu_{1}}P_{1}^{l}+\sum_{l=1}^{\mu_{2}}P_{2}^{l}+\cdots +\sum_{l=1}^{\mu_{j}}P_{j}^{l}+\cdots$$ (2)

where $S_{i}^{k}$ is the *k-*th molecule of *S*_*i*_, and $P_{j}^{l}$ is the *l*-th molecule of *P*_*j*_. $S_{i}^{k}$ and $P_{j}^{l}$ can be further represented as:

$$\begin{array}{c} S_{i}^{k}\to S_{i}^{k}\{n(S_{i}^{k}\backslash P_{j}^{l}),\;n(S_{i}^{k}⋂P_{j}^{l})\}\\ P_{j}^{l}\to P_{j}^{l}\{n(P_{j}^{l}\backslash S_{i}^{k}),\;n(S_{i}^{k}⋂P_{j}^{l})\} \end{array}$$ (3)

where $n(S_{i}^{k}\backslash P_{j}^{l})$ represents the number of atoms belonging to $S_{i}^{k}$ but not transferred to $P_{j}^{l},n(P_{j}^{l}\backslash S_{i}^{k})$ suggests the number of atoms belonging to $P_{j}^{l}$ but not derived from $S_{i}^{k}$, and $n(S_{i}^{k}⋂P_{j}^{l})$ indicates the number of atoms simultaneously mapped to $S_{i}^{k}$ and $P_{j}^{l}$. The similarity between $S_{i}^{k}$ and $P_{j}^{l}$ can be expressed as Tversky index:

$$T_{Sik\_Pjl}=\frac{n(S_{i}^{k}⋂P_{j}^{l})}{\alpha ∙n(S_{i}^{k}\setminus P_{j}^{l})+\beta ∙n(P_{j}^{l}\setminus S_{i}^{k})+n(S_{i}^{k}⋂P_{j}^{l})}\;0\leq \alpha ,\beta \leq 1$$ (4)

In particular, it equals to Tanimoto index when α = β = 1, and equals to Dice index when α = β = 0.5. Finally, the uniform similarity between substrate *S*_*i*_ and product *P*_*j*_ can be formulated as:

$$T_{Si\_Pj}=\frac{\sum_{k=1}^{\lambda_{i}}\sum_{l=1}^{\mu_{j}}T_{Sik\_Pjl}}{\sum_{k=1}^{\lambda_{i}}\sum_{l=1}^{\mu_{j}}\left\{m|m=1\;if\;T_{Sik\_Pjl}\neq 0,\;m=0\;if\;T_{Sik\_Pjl}=0\right\}}$$ (5)

The uniform similarity can effectively avoid the influence of different stoichiometric coefficients in biochemical reactions, for instance extended reactions. In order to accurately identify the main substrate-product pairs in biochemical reactions, we exclude atoms mapped to GC before calculating the similarity. Besides, we also found that by excluding atoms affiliated to three specific substructures (namely CoA group, Pi group and PPi group), the quality of substrate-product pairs could be further improved. After eliminating atoms belonging to GC and specific substructures, Eq (4) can be rewritten as:

$$T_{Sik\_Pjl}=\frac{n(S_{i}^{k}⋂P_{j}^{l}\backslash E_{x})}{\alpha ∙n(S_{i}^{k}\setminus P_{j}^{l}\backslash E_{x})+\beta ∙n(P_{j}^{l}\setminus S_{i}^{k}\backslash E_{x})+n(S_{i}^{k}⋂P_{j}^{l}\backslash E_{x})}\;0\leq \alpha ,\beta \leq 1$$ (6)

where *E*_*x*_ is a pseudo molecule, which contains all atoms mapped to the GC and specific substructures. In addition, in order to highlight the intersection of $S_{i}^{k}$ and $P_{j}^{l}$ in the generation of substrate-product pairs, Dice index was used.

#### Calculation of main substrate-product pairs.

Uniform similarity is utilized to generate substrate-product pairs. In the calculation, we can choose the atom types to be counted (e.g. C, H, O, N and P) and the types of atom transfer to be satisfied (e.g. C). For example, substrate-product pairs without carbon atom transfer are abandoned.

In the biochemical reaction *R*_*X*_, all products *P*_*j*_ that meet the following inequality are the main products of the substrate *S*_*i*_:

$$|T_{Si}^{max}-T_{Si\_Pj}|\leq \varepsilon$$ (7)

where, $T_{Si}^{max}=max\{T_{Si\_Pj},\forall j\},0\leq \varepsilon \leq 1$.

Similarly, all substrates *S*_*i*_ that meet the following inequality are the main substrates of the product *P*_*j*_:

$$|T_{Pj}^{max}-T_{Si\_Pj}|\leq \varepsilon$$ (8)

where, $T_{Pj}^{max}=max\{T_{Si\_Pj},\forall i\},0\leq \varepsilon \leq 1$.

When these two inequalities are simultaneously satisfied, the substrate *S*_*i*_ and product *P*_*j*_ constitute one main substrate-product pair of biochemical reaction *R*_*X*_. Ultimately, the main substrate-product pairs generated from all biochemical reactions constitute the EMN that used for subsequent pathway design.

### Conditional search method

**Fig 3:**
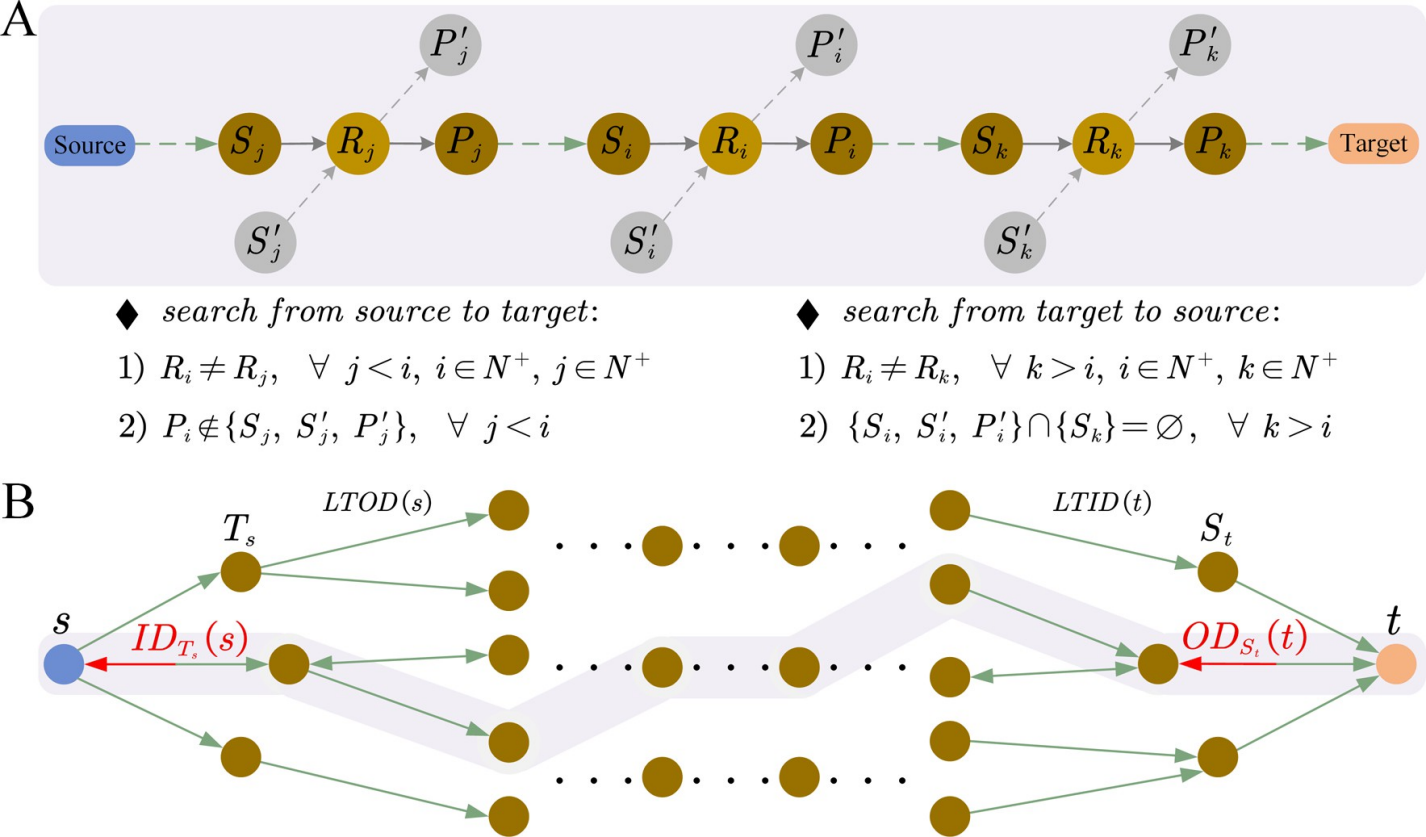
Illustration of the CSS. (A) Two constraints that biologically feasible and linear metabolic pathways need to meet. The two constraints are labeled as ‘1)’ and ‘2)’. The green dashed arrow means multi-step reactions and the gray dashed arrow indicates implicit access of compound nodes (substrates or products). (B) CSS based on local total in-out degree (LTIOD). *LTOD*(*s*) indicates the local total out degree of initial substrate and *LTID*(*t*) denotes the local total in degree of target product. Forward search, from initial substrate (Source) to target product (Target). Reverse search, from target product (Target) to initial substrate (Source).

A biologically meaningful and linear metabolic pathway is a simple pathway, containing no loops, and needs to satisfy two constraints as shown in Fig 3A, in which *S*_*i*_ and $S_{i}^{\prime }$ are the substrates, *P*_*i*_ and $P_{i}^{\prime }$ are the products of biochemical reaction *R*_*i*_, respectively. Constraint 1 means that each biochemical reaction can only occur once in one metabolic pathway. Constraint 2 indicates that each important intermediate metabolite cannot be identical to the substrate or product of the upstream biochemical reactions.

When forward search is applied, constraint 2 is equivalent to:

$$P_{i}\notin \{S_{1},S_{1}^{\prime },P_{1}^{\prime },\cdots ,S_{i-1},S_{i-1}^{\prime },P_{i-1}^{\prime }\}$$ (9)

On the contrary, when reverse search is employed, constraint 2 is equivalent to:

$$\{S_{i},S_{i}^{\prime },P_{i}^{\prime }\}\cap \{S_{i+2},S_{i+3},\cdots ,\}=\emptyset$$ (10)

Constraint 1 and constraint 2 ensure that there are no loops composed of main intermediate metabolites in one metabolic pathway, while they also complicate the pathway search problem. For example, even if two nodes (such as lr_R01978 and rl_R01978) were used to represent the different directions of reversible reaction R01978, they could not appear in the same metabolic pathway at the same time. Furthermore, the implicit access of compound nodes in the metabolic network (gray nodes in Fig 3A) is more complicated.

In this paper, it was found that the structure of metabolic network was obviously imbalanced which mainly reflected in the complexity of primary metabolism and the relative simplicity of secondary metabolism (S1A Fig). More interestingly, the average search time from primary metabolite to secondary metabolite proved to be significantly higher than that from secondary metabolite to primary metabolite (S1B Fig), which suggests making full use of the imbalance to effectively shorten the search time. Based on these discoveries, we propose a CSS based on LTIOD, as described in Fig 3B. In order to correlate the imbalance of network structure with the search time, we define the local total out degree of initial substrate as *LTOD*(*s*) and the local total in degree of target product as *LTID*(*t*), respectively:

$$LTOD(s)=\sum_{c_{i}\in T_{s}}OD(c_{i})-{ID}_{T_{s}}(s)$$ (11)

$$LTID(t)=\sum_{c_{i}\in S_{t}}ID(c_{i})-{OD}_{S_{t}}(t)$$ (12)

where *T*_*s*_ represents the product set of initial substrate *s*, *OD*(*c*_*i*_) indicates the out-degree of compound *c*_*i*_, ${ID}_{T_{s}}(s)$ denotes the in-degree of initial substrate *s* derived from its product set *T*_*s*_; *S*_*t*_ represents the substrate set of target product *t*, *ID*(*c*_*i*_) indicates the in-degree of the compound *c*_*i*_, and ${OD}_{S_{t}}(t)$ denotes the out-degree of target product *t* to its substrate set *S*_*t*_.

According to these definitions, *LTOD*(*s*) and *LTID*(*t*) could be compared to select an optimal search direction with the smaller one, that is, when *LTOD*(*s*)≤*LTID*(*t*) forward search should be selected, otherwise reverse search should be selected. It should be pointed out that the extension process in pathway search (both forward or reverse search) should meet the two constraints shown in Fig 3A, and that the search strategy is applied only once to the initial substrate and the target product at the beginning of a search to help decide the search direction.

The combination of CSS based on LTIOD and breadth-first search method is validated to solve most search problems of metabolic pathway, such as the substrate-missing pathway (S2 Fig), the given-step pathway (S3 Fig) and the shortest pathway (S4 Fig). However, the breadth-first search method is not always effective, especially when both the initial substrate and the target product are primary metabolites, such as the biosynthetic pathway of L-histidine. Due to the high complexity of the local network structure around primary metabolites, it produces a large number of intermediate results. So as to address this limitation in certain scenarios, we also combine the LTIOD-based CSS with depth-first search method to expand the applicable scope of PyMiner. In addition, the pseudo codes of CSS are provided in S2 File.

### Comprehensive evaluation method

Considering the complexity of metabolic network structure, a large number of metabolic pathways could be retrieved. As a result, it is critical to evaluate and rank the retrieved metabolic pathway candidates. Here, we established an exquisite set of evaluation indexes, including infeasible pathway length (*l*_*inf*_), exogenous pathway length (*l*_*exo*_), endogenous pathway length (*l*_*endo*_), substrate-atom utilization (*S*_*uti*_), product-atom conservation (*T*_*con*_), and main metabolic flux (*v*_*target*_) of target product, while the priority of these six indexes decreases in sequence. Using the six indexes with different priority orders, we could evaluate and rank all the retrieved metabolic pathways. Specifically, we prefer the metabolic pathway with shorter length of the infeasible pathway, and when this index is the same, we prefer the metabolic pathway with shorter length of the exogenous pathway, the rest can be done in the same manner. Finally, the ordered results of the retrieved metabolic pathways and their corresponding indexes were given by PyMiner. In addition, the pseudo codes of metabolic pathway evaluation are provided in S2 File.

Given the genome-scale metabolic network model (GSMM) of one chassis microorganism, the length of endogenous steps (*l*_*endo*_), exogenous steps (*l*_*exo*_) and infeasible steps (*l*_*inf*_) are counted for each metabolic pathway. According to the mapping relationship between the reactions as well as compounds in the EMN and the reactions as well as metabolites in the GSMM, we first counted all the endogenous or exogenous reactions, endogenous or exogenous compounds and infeasible reactions. And the infeasible reactions mainly refer to the exogenous reactions whose substrates are partially missing in the chassis microorganism. For example, the construction of the synthesis pathway for ethylene glycol in *Escherichia coli* requires adding xylose to the culture medium [14, 20]. The GSMMs of commonly used model microorganisms, including *Escherichia coli* (eco), *Saccharomyces cerevisiae* (sce), *cyanobacteria* (syz), and so on, were derived from BIGG database [21] and integrated into PyMiner. Subsequently, PyMiner managed to give higher priority to the metabolic pathways with shorter *l*_*endo*_, *l*_*exo*_ and *l*_*inf*_.

According to the atom mapping information, the previously calculated uniform similarity can only guarantee a high atom utilization of substrate and a high atom conservation of product in single-step reaction other than the entire pathway. As a consequence, we tried to conceive global evaluation indexes by tracing the atom transfer route across the entire metabolic pathway. Consistent with the calculation of substrate-product similarity, we only traced the transfer route of non-hydrogen and key atoms, and excluded the atoms belonging to GC and specific substructures. The atom utilization of the initial substrate and the atom conservation of the target product in a specified metabolic pathway is given by:

$$S_{uti}=\frac{N_{A}(source\cap target)}{N_{A}(source)}$$ (13)

$$T_{con}=\frac{N_{A}(source\cap target)}{N_{A}(target)}$$ (14)

where *N*_*A*_(*source*∩*target*) represents the number of atoms that simultaneously mapped to the initial substrate and the target product, *N*_*A*_(*source*) and *N*_*A*_(*target*) indicate the number of atoms in the initial substrate and the target product, respectively. It is important to note that since only atoms within a single molecule were traced, then if the stoichiometric coefficient *λ*_*i*_ or *μ*_*j*_ does not equal to one, the calculated results of Eqs (13) and (14) may be slightly different from their true values. Subsequently, we delivered higher priority to the metabolic pathways with a higher *S*_*uti*_ and *T*_*con*_, and further excluded those pathways without atom transfer from the initial substrate to the final product.

Metabolic flux, that is, the maximum synthesis rate of the target product, is another important index to quantify the production capacity of one metabolic pathway. Given the GSMM of a specific chassis microorganism, we employed the flux balance analysis tool namely COBRApy [22] to calculate the maximum metabolic flux *ν*_*target*_. The optimization problem [23] can be formulated as:

$$\begin{array}{l} \max\;v_{target}=c^{T}\nu \\ s.t.\;\sum_{j=1}^{N}S_{ij}\upsilon_{j}=0,\;i=1,2,\cdots ,M\\ \;\upsilon_{j}^{l}\leq \upsilon_{j}\leq \upsilon_{j}^{u},\;j=1,2,\cdots ,N\\ \;\nu_{biomass}\geq \eta \nu_{biomass}^{max},\;0<\eta <1 \end{array}$$ (15)

where, ***c*** is a binary vector with one ‘1’ and the position of ‘1’ is corresponding to the excretion rate of target product, *S*_*ij*_ indicates the stoichiometric coefficient of metabolite *i* in the metabolic reaction *j*, *υ*_*j*_ is the metabolic flux of reaction *j*, M and N denote the number of metabolites and reactions, $\upsilon_{j}^{l}$ and $\upsilon_{j}^{u}$ mean the lower and upper bound of metabolic flux *υ*_*j*_, respectively. *ν*_*biomass*_ equals to the biomass synthesis rate of engineered bacteria, while $\nu_{biomass}^{max}$ represents the maximum biomass synthesis rate of wild-type strain. By setting the pre-defined value of *η* (e.g. 0.8), a minimum growth rate of the chassis microorganism can be maintained.

The theoretical synthetic rate *ν*_*target*_ obtained from Eq (15) can reveal the differences of diverse metabolic pathways that are entirely composed of exogenous reactions. However, when metabolic pathways contain both endogenous and exogenous reactions, *ν*_*target*_ does not necessarily show differences in results, especially when the retrieved endogenous part is different, but the retrieved exogenous part is the same. Since the same exogenous portion of the metabolic pathways makes no difference among GSMMs of one chassis microorganism even after fusing different metabolic pathways, so the maximum synthesis rate *ν*_*target*_ acquired from Eq (15) is identical.

In order to address this problem, we put forward a method to calculate the main metabolic flux. As shown in S5A Fig, we assume that *C*_*i*_, *C*_*j*_ and *C*_*k*_ are examples of the main intermediate metabolites, which are mainly formed by the substrates *A*_*i*_/*B*_*i*_, *A*_*i*_/*B*_*j*_, and *A*_*k*_/*B*_*k*_ respectively. This hypothesis suggests that the main intermediate metabolites and target product are mainly produced by the reactions (such as *R*_*i*1_, *R*_*j*1_ *and R*_*k*1_) in the metabolic pathway instead of other endogenous reactions (such as *R*_*i*2_, *R*_*j*2_ and *R*_*k*2_) in the chassis microorganism. Therefore, the metabolic fluxes with respect to metabolite *C*_*i*_ satisfy the constraints ($v_{i1}\geq v_{i2},v_{i1}\geq v_{i3},\cdots ,v_{i1}\geq v_{iW_{i}}$) shown in S5B Fig. However, these additional constraints can’t be introduced into one GSMM directly by using COBRApy. Through adding additional pseudo metabolites ($M_{t2},M_{t3},\cdots ,M_{tW_{i}}$) and pseudo reactions ($R_{t2},R_{t3},\cdots ,R_{tW_{i}}$) by using COBRApy, the metabolic flux constraints shown in S5B Fig can be transformed into the equivalent constraints shown in S5C Fig, that is, $v_{t2}\geq 0,v_{t3}\geq 0,\cdots ,v_{tW_{i}}\geq 0$. Similar constraints are introduced to all main intermediate metabolites (such as *C*_*i*_, *C*_*j*_ and *C*_*k*_) in one pathway. With additional information to the GSMM, we can utilize COBRApy to calculate the main metabolic flux *ν*_*target*_ of the metabolic pathway with respect to the target product. This calculation method can be appropriate for the scenario where the metabolic fluxes of different metabolic pathways are identical due to the same exogenous parts.

## Results

### The property of the extracted metabolic network

In this study, Dice index was employed to calculate the uniform similarity between substrate and product. To study the influence of diverse similarity difference thresholds ε on the construction of EMN, we set the value of ε to 10 equi-spaced levels (0.1, 0.2, …, 1.0), and generated corresponding substrate-product pairs respectively. Compared with GMN, significant decrease levels in the number of substrate-product pairs are found at ε = 0.1, namely, 79.63% (KEGG), 82.94% (MetaCyc) and 82.28% (KndPad) (S6 Fig). Therefore, redundant information contained in GMN is removed, and the number of substrate-product pairs are shown to decrease by 81.62% on average. For example, when ε = 0.1, the substrate-product pairs produced by reversible reaction R01978 from KEGG were C00356_C00332 and C00332_C00356. In contrast, when ε = 0.3, the pairs produced by the same reaction were C00356_C00024, C00024_C00356, C00356_C00332 and C00332_C00356, while the former two are not the main substrate-product pairs of reaction R01978. Especially, when ε = 1.0, all substrates containing transferred carbon atom along with their products constituted the main substrate-product pairs. In this example, it was observed that by strengthening the similarity constraint with lower thresholds ε on the candidates, the quality of substrate-product pairs could be improved.

### Performance evaluation of PyMiner

**Fig 4:**
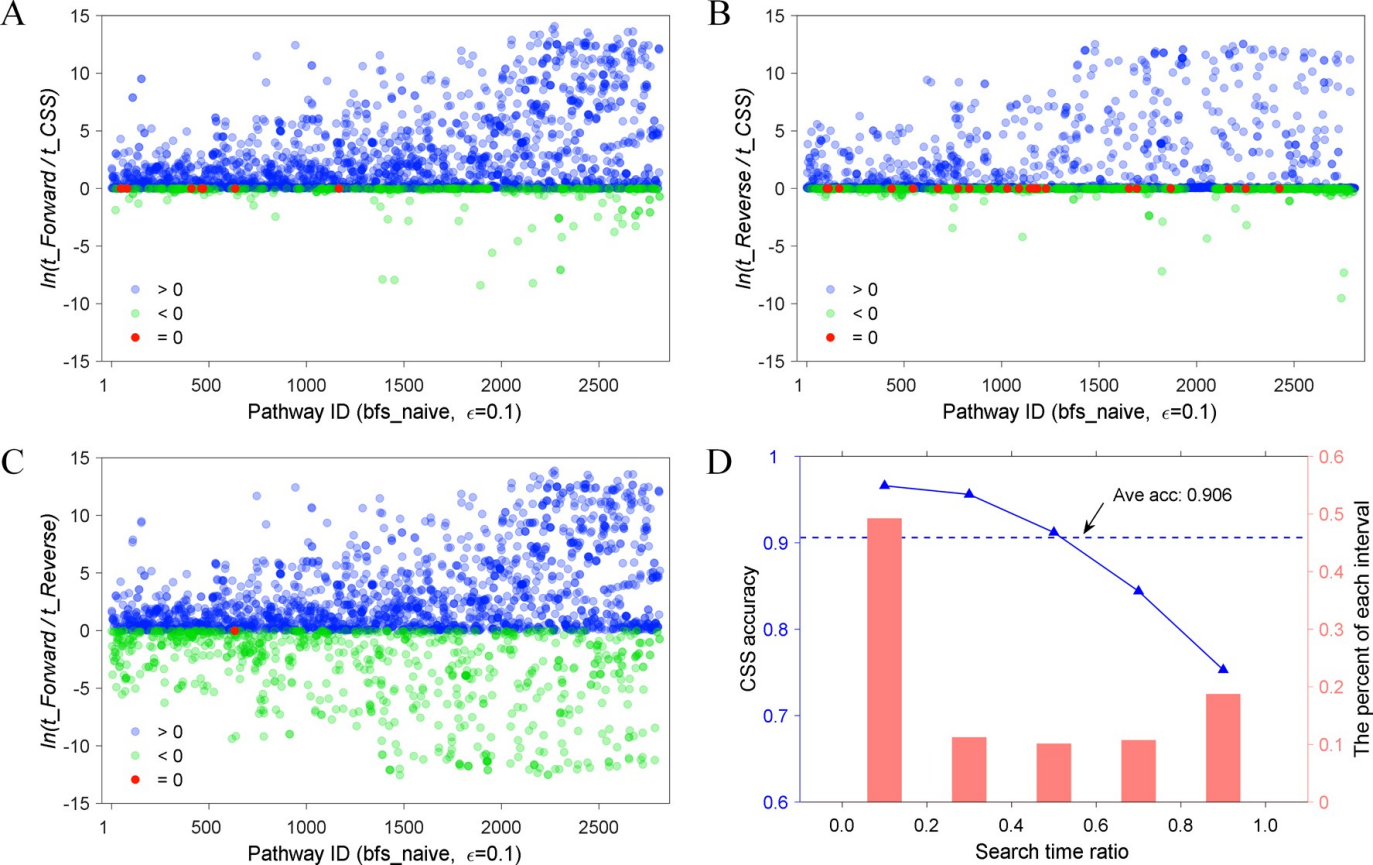
Comparative performance of time consumption on different search strategies. (A) The scatter plots of time consumption ratio of forward search divided by CSS based on LTIOD. (B) The scatter plots of time consumption ratio of reverse search divided by CSS based on LTIOD. (C) The scatter plots of time consumption ratio of forward search divided by that of reverse search. The horizontal and vertical axes of panels A, B and C are pathways ID and the natural logarithmic ratio of time consumption, respectively. The time consumption ratio is shown in natural logarithms. Blue, green and red dots represent ratio greater than, less than or equal to zero, respectively. Bfs_naive indicates breadth-first search method. (D) The accuracy and proportion distribution of LTIOD-based CSS on different intervals of search time ratio.

We first evaluated pathway search performance of different search strategies on the validation set with 2812 linear pathways. To be more specific, comparative studies among traditional forward search strategy, reverse search strategy, and the proposed LTIOD-based CSS were performed to search for each pathway in the validation set and the corresponding search times were recorded. The scatter plots of comparative results are demonstrated in Fig 4A–4C. Obviously, the LTIOD-based CSS has enormous advantages in search time (Fig 4A and 4B) as 84.3% and 66.6% cases exhibiting less time consumption (blue dots) relative to forward search and reverse search, respectively. Meanwhile, the reverse search strategy is superior to the forward search strategy in 71.5% cases, which may attribute to that a large number of target products are secondary metabolites in the validation set (40.7%). To further evaluate the accuracy, the ratio of the smaller forward (or reverse) search time to the larger reverse (or forward) search time was divided into different intervals, and the statistical distribution of the accuracy of the LTIOD-based CSS was obtained (Fig 4D). The correct criterion is that if the CSS is consistent with the less time-consuming forward or reverse search strategy, *vice versa*. In total, CSS performed less time-consuming search on 2549 out 2812 Pathways. Therefore, the average accuracy of CSS is 90.6% (2549/2812). The statistical results in Fig 4D also clearly show that the greater difference between forward and reverse search time (e.g. the smaller ratio in range 0 to 0.2) leads to the higher accuracy of the LTIOD-based CSS, which may result from the structure imbalance of metabolic network (S1A Fig). If ε increases to 1.0, compared to ε = 0.1, the numbers of substrate-product pairs are shown to increase 22.2% on average (S6 Fig). As a result, the average search times of forward search strategy, reverse search strategy and LTIOD-based CSS increase. However, considering that the EMN is imbalanced, the search strategy (CSS) proposed in this study still works, despite that the logarithmic ratios of time consumption in some cases increase while in other cases decrease. Therefore, the CSS based on LTIOD were applied and integrated into the PyMiner for metabolic pathway search.

**Fig 5:**
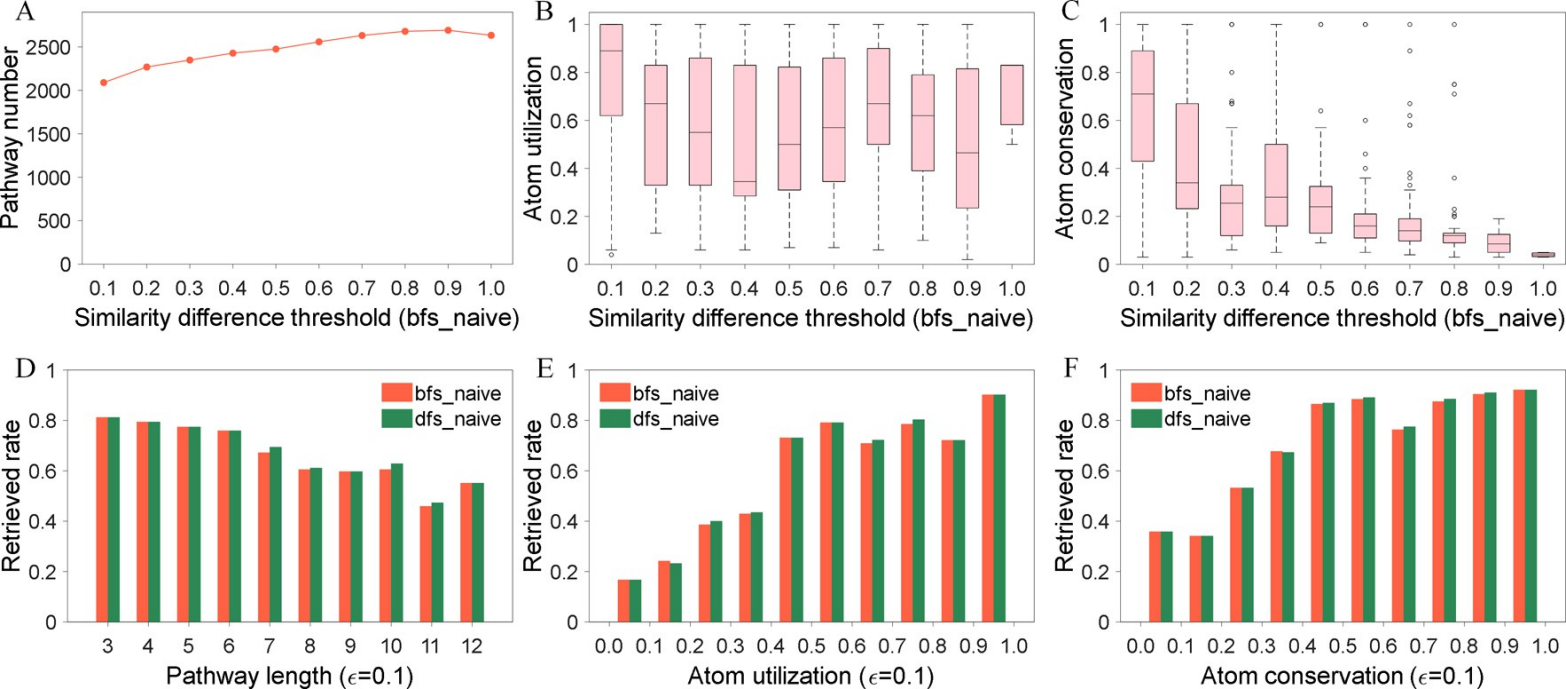
Relationships between the search results of metabolic pathways in the validation set and the pivotal parameters of PyMiner, including similarity difference threshold, pathway length, atom utilization and conservation. (A) The effect of similarity difference threshold ε on the number of retrieved pathways. (B) The effect of similarity difference threshold ε on the atom utilization of initial substrate. (C) The effect of similarity difference threshold ε on the atom conservation of target product. (D) The relationship between pathway length and the retrieved rate. (E) The relationship between the atom utilization of initial substrate and the retrieved rate. (F) The relationship between the atom conservation of target product and the retrieved rate. Bfs_naive indicates breadth-first search method. Dfs_naive denotes depth-first search method. Due to the constraints of maximum search time and computing resources, the search results of two methods show slight differences.

Based on the uniform similarity between substrate and product, the candidate pathways with high utilization of substrate atoms and high conservation of product atoms should be preferentially extracted by PyMiner. We also explored the influence of key variable of PyMiner, namely similarity difference threshold ε, on the search results of metabolic pathway. Fig 5A–5C statistically depicted various parameter results of the retrieved metabolic pathways at a given threshold ε. Retrieved pathways that found at a smaller value than the pre-set ε were excluded, and only new additions were counted. With the gradual increase of threshold ε, the number of retrieved metabolic pathways increases slowly (Fig 5A), and the product-atom conservation decreases gradually (Fig 5C), which is consistent with the original design intention. However, the variation trend of substrate-atom utilization is not obvious (Fig 5B). This is because we excluded atoms belonging to GC and specific substructures in tracing the atom transfer route and calculating the initial substrate atom utilization, and that compressed the space of further loss of initial substrate atoms along the pathway. However, when ε = 0.1, the utilization of substrate atoms and the conservation of product atoms are significantly higher than that of other values of ε. Therefore, a smaller ε value (such as 0.1) could be preferentially selected in the application of PyMiner.

In addition to the influence of ε, we further analyzed the relationship between different evaluation indexes, involving metabolic pathway length, substrate-atom utilization, product-atom conservation and the retrieved rate (the percent of pathways successfully retrieved by PyMiner) of metabolic pathway (see details in Fig 5D–5F). It is easy to learn from Fig 5D that the metabolic pathway with longer length is harder to search for, which is consistent with our intuitive understanding. At the same time, the higher utilization of substrate atoms or the higher conservation of product atoms leads to the greater probability that this metabolic pathway will be retrieved (Fig 5E and 5F), which coincides with our original intention of the ranking priority of metabolic pathways.

### Comparison with reported methods for metabolic pathway design

We summarized several representative methods for metabolic pathway design, and concluded their characteristics in Table 1. Here, we systematically compared PyMiner method with four state-of-the-art methods (RouteSearch, MRE, EcoSynther and PATH^cre8^) from various perspectives. As depicted in Table 3, we demonstrated 20 representative metabolic pathways that were experimentally verified, where nearly half (9/20, S3 Table) of these pathways came from the compared methods (such as RouteSearch, MRE and PATH^cre8^), and the remaining 11 pathways were derived from a comprehensive review article [1]. These metabolic pathways have a long span in length (2–13 steps) and a wide distribution range (including industrial chemicals and natural chemicals) to meet the requirement of sufficient representation. In addition, the pathways of which the corresponding GSMMs are not available from BIGG [21] were not included. Table 3 also exhibits the length of the endogenous and exogenous steps of each metabolic pathway in the given chassis microorganism, and the retrieved results of various search methods. It could be found from Table 3 that PyMiner has definite advantages as its rank performance is equal or better than other methods on 95% (19/20, except for the biosynthesis pathway of violacein) of cases. In details, in some cases, only PyMiner could retrieve the corresponding pathways; in other cases, the corresponding pathways retrieved by PyMiner have higher rankings. PyMiner integrates more biochemical reaction data and expands the space of pathway search. By constructing GC and calculating the uniform similarity between substrate and product, PyMiner further excludes a large number of substrate-product pairs with low atom-transfer rate. Furthermore, PyMiner selects biologically meaningful metabolic pathways by using exquisite evaluation indexes such as pathway length, substrate-atom utilization, product-atom conservation and main metabolic flux. Table 4 comprehensively demonstrates the evaluation indexes of these metabolic pathways (see more details in S3 Table). In addition, we also compared PyMiner with other four methods on the validation sets and the databases (e.g. KEGG and MetaCyc) they applied, including 5 cases for RouteSearch, 8 cases for MRE, 2 cases for EcoSynther and 20 cases for PATH^cre8^, and the corresponding results again confirmed that our method still maintained an edge. In details, PyMiner performs better or equally on 100% (5/5), 87.5% (7/8), 100% (2/2) and 75% (15/20) of cases, respectively (see more details in S4 Table). The poor performance on the validation set of PATH^cre8^ is due to the exclusion of inessential substrate-product pairs.

**Table 3:** Experimentally validated biosynthesis pathways retrieved by PyMiner and other methods.

Source	Target	Host	Endo/exogenous steps	Pathway Rank					(Ref.)
				PyMiner	RouteSearch	MRE	EcoSynther	PATH^cre8^	
glycerol	(R)-propane-1,2-diol	eco	5/0	1	—	No	—	10	[24]
glycerol	1,3-propanediol	eco	0/2, 0/2	1, 2	—	1, No	1, 2	1, No	[25]
glycerol	3-hydroxypropionate	eco	0/2	1	—	No	No	No	[26]
pyruvate	isopropanol	eco	3/2	3	—	32	—	No	[27]
pyruvate	isobutanol	**eco**	**3/2**	**1**	**1**	**No**	**No**	**No**	[28]
L-tyrosine	umbelliferone	eco	0/4	1	1	No	No	No	[29]
L-tyrosine	(2S)-naringenin	eco	0/4	1	—	1	No	1	[30]
L-tyrosine	trans-resveratrol	eco	0/3	1	—	1	Top *x*	1	[31]
L-tryptophan	violacein	eco	0/5	3*	—	No	No	1	[32]
erythrose-4P	cis,cis-muconate	eco	3/3	1	—	1	—	No	[33]
glycerol	(R)-propane-1,2-diol	sce	3/2, 3/2	1, 3*	—	7, 6	—	9, 7	[34]
pyruvate	2,3-butanediol	sce	2/1	1	—	1	—	3	[35]
L-phenylalanine	(2S)-naringenin	sce	1/4	2	—	2	—	No	[36]
acetyl-CoA	artemisinate	sce	8/2	1	—	91	—	No	[37]
acetyl-CoA	taxa-4,11-diene	**sce**	**9/1**	**1**	**—**	**3, 45**	**—**	**No**	[38]
acetyl-CoA	all-trans-lycopene	sce	9/2	1	—	1, 39	—	No	[39]
acetyl-CoA	glycyrrhetinate	sce	10/3	1	—	3, 4	—	No	[40]
L-phenylalanine	(2S)-pinocembrin	**sce**	**0/4**	**1**	**—**	**1**	**—**	**2**	[41]
pyruvate	isoprene	syz	5/3	2	—	228	—	No	[42]
acetyl-CoA	isoprene	syz	2/6	1*	—	11	—	No	[42]

*Note*: 1* means tying for first place, and 3* means tying for third place. Top *x* means that the rank of the corresponding pathway is uncertain. The solid line indicates missing value due to various reasons, including unavailable host organism, primary database and default source set. Organism names are: eco, *Escherichia coli K-12 MG1655*; sce, *Saccharomyces cerevisiae S288c*; and syz, *Synechocystis sp*. *PCC 6803*. Default input values were used in all examples unless otherwise stated (detailed in S3 Table). Specifically, pathway length was set to accommodate known pathways.

**Table 4:** Detailed metrics of the biosynthetic pathways discovered by PyMiner.

Source	Target	Total Length	Infeasible Length	Exogenous Length	Endogenous Length	Atom Utilization	Atom Conservation	Metabolic Flux (mmol gDW^-1^ hr^-1^)
glycerol	(R)-propane-1,2-diol	5	0	0	5	0.83	1.0	2.65
glycerol	1,3-propanediol	2, 2	0, 0	2, 2	0, 0	0.83, 0.83	1.0, 1.0	2.76, 2.71
glycerol	3-hydroxypropionate	2	0	2	0	0.83	0.83	2.98
pyruvate	isopropanol	5	0	2	3	0.5	0.75	2.90
pyruvate	isobutanol	**5**	**0**	**2**	**3**	**0.5**	**0.6**	**2.08**
L-tyrosine	umbelliferone	4	0	4	0	0.85	0.92	1.04
L-tyrosine	(2S)-naringenin	4	0	4	0	0.85	0.55	0.69
L-tyrosine	trans-resveratrol	3	0	3	0	0.77	0.59	0.69
L-tryptophan	violacein	5	0	5	0	0.87	0.5	0.45
erythrose-4P	cis,cis-muconate	6	0	3	3	0.75	0.6	1.66
glycerol	(R)-propane-1,2-diol	5, 5	0, 0	2, 2	3, 3	0.83, 0.83	1.0, 1.0	2.88, 2.54
pyruvate	2,3-butanediol	3	0	1	2	0.5	0.5	2.42
L-phenylalanine	(2S)-naringenin	5	0	4	1	0.83	0.5	0.60
acetyl-CoA	artemisinate	10	0	2	8	0.67	0.12	0.47
acetyl-CoA	taxa-4,11-diene	**10**	**0**	**1**	**9**	**0.67**	**0.1**	**0.35**
acetyl-CoA	all-trans-lycopene	11	0	2	9	0.67	0.05	0.17
acetyl-CoA	glycyrrhetinate	13	0	3	10	0.67	0.06	0.23
L-phenylalanine	(2S)-pinocembrin	**4**	**0**	**4**	**0**	**0.83**	**0.53**	**0.59**
pyruvate	isoprene	8	0	3	5	0.33	0.4	1.75
acetyl-CoA	isoprene	8	0	6	2	0.67	0.4	1.75

*Note*: The priority of these six metrics of each retrieved pathway descends from left to right.

#### Biosynthesis of 1,3-propanediol and 1,2-propanediol.

Both 1,3-propanediol and 1,2-propanediol are important chemical raw materials, which are mainly used as monomers in polyester synthesis [1]. As displayed in Table 3, PyMiner method retrieved two metabolic pathways for *Escherichia coli* to synthesize 1,3-propanediol from glycerol. The main difference lies in the cofactors used in the last biochemical reactions, namely NADH and NADPH [25]. Between these two pathways, the main metabolic flux of the target product in the former is slightly dominant (row 2 in Table 4). Interestingly, the maximum synthesis rate of NADH is also slightly superior compared with NADPH (0.51 vs 0.50). In addition, PyMiner also discovered two metabolic pathways for *Saccharomyces cerevisiae* to synthesize 1,2-propanediol from glycerol, which share the same exogenous part but different endogenous part (row 11 in Table 4). One metabolic pathway uses cofactor FADH2 to directly transfer electrons to the mitochondrial respiratory chain, and the other pathway uses NADPH as cofactor which mainly occurs in cytoplasm, in agreement with previous study [34]. This result has suggested that if glucose instead of glycerol is fed as the sole carbon source, the first metabolic pathway seems to be a better choice to synthesize 1,2-propanediol. However, when the traditional flux balance analysis (without adding additional constraints to the metabolic flux corresponding to the intermediates metabolite or target product of one pathway) is applied, it is difficult to distinguish the differences of these two metabolic pathways, which also shows the advantages of the main metabolic flux calculation method in PyMiner.

#### Biosynthesis of isobutanol and pinocembrin.

**Fig 6:**
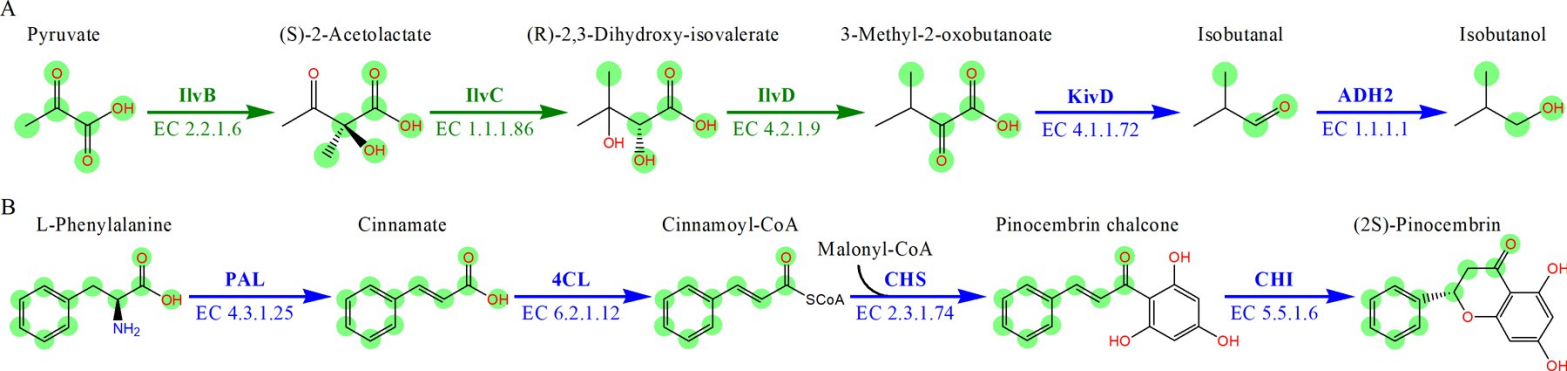
The top-ranked biosynthesis pathways of isobutanol and pinocembrin retrieved by PyMiner. (A) The biosynthetic pathway to produce isobutanol from pyruvate in E. coli. (B) The biosynthetic pathway to produce pinocembrin from L-phenylalanine in S. cerevisiae. The green arrows indicate endogenous steps while the blue arrows denote exogenous steps. The green circles highlight the atom transfer route from the initial substrate to the target product. And the EC number of each step is indicated below the arrow. Enzyme names are: IlvB, acetolactate synthase; IlvC, ketol-acid reductoisomerase; IlvD, dihydroxy-acid dehydratase; KivD, α-ketoisovalerate decarboxylase; ADH2, alcohol dehydrogenase; PAL, phenylalanine ammonia lyase; 4CL, cinnamoyl-CoA ligase; CHS, chalcone synthase; and CHI, chalcone isomerase.

Isobutanol is an important industrial solvent and gasoline additive [1]. The reported synthetic pathway of isobutanol from pyruvate that constructed in *E*. *coli* consists of 3-step endogenous reactions and 2-step exogenous reactions [28]. PyMiner was employed to retrieve the isobutanol biosynthesis pathway with pyruvate as the initial substrate, and the reported 5-step synthesis pathway was accurately retrieved and ranked first in the search results (row 5 in Table 3). RouteSearch also retrieved this synthetic pathway, but the other three methods couldn’t find this pathway. More importantly, PyMiner also traced and highlighted the transfer route of key atoms in this isobutanol synthesis pathway with green markers shown in Fig 6A. The graphical results indicated that three key atoms originated from pyruvate were transferred to the target product isobutanol. The key atom utilization of pyruvate and the key atom conservation of isobutanol were 0.5 and 0.6 (row 5 in Table 4), respectively, and identical to their true values.

Pinocembrin is a natural flavonoid with a high medicinal and economic value for its derivatives [41]. The synthetic pathway of pinocembrin constructed in *S*. *cerevisiae* is entirely composed of exogenous biochemical reactions as shown in Fig 6B. PyMiner, MRE, and PATH^cre8^ successfully searched for this 4-step synthetic pathway of pinocembrin from L-phenylalanine (18 row in Table 3). Due to the superiority of PyMiner in calculating the similarity between substrate and product, even although nearly half (47%) of the key atoms of pinocembrin chalcone come from malonyl coenzyme A, PyMiner could still excluded it from the main substrate-product pairs of the third-step reaction. The corresponding key atom utilization of L-phenylalanine and key atom conservation of pinocembrin were 0.83 and 0.53 (18 row in Table 4), respectively, consistent with the true values.

#### Biosynthesis of taxadiene.

**Fig 7:**
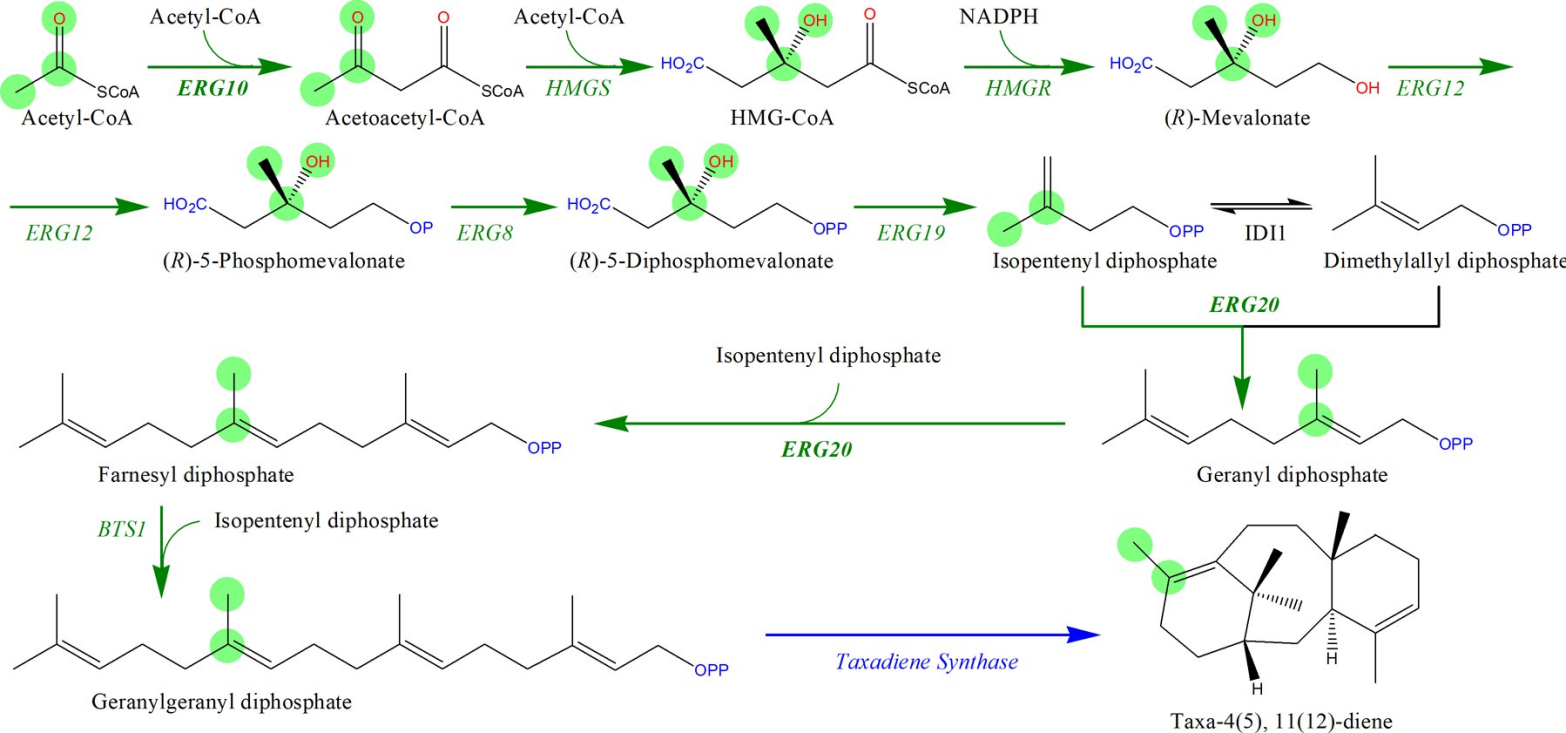
The experimentally validated biosynthesis pathway of taxadiene from acetyl-CoA in *S*. *cerevisiae* retrieved by PyMiner. The green arrows indicate endogenous steps while the blue arrows denote exogenous steps. The green circles highlight the atom transfer route from acetyl-CoA to taxadiene. The abbreviations are: ERG10, acetyl-CoA acetyltransferase; HMGS, HMG-CoA synthase; HMGR, HMG-CoA reductase; ERG12, mevalonate kinase; ERG8, phosphomevalonate kinase; ERG19, mevalonate diphosphate decarboxylase; ERG20, farnesyl diphosphate synthase; and BTS1, geranylgeranyl diphosphate synthase.

Taxadiene is a crucial intermediate metabolite in the biosynthetic pathway of paclitaxel. As an important anti-cancer drug, paclitaxel is widely applied in the treatment of ovarian cancer, breast cancer and non-small cell lung cancer [43]. As shown in Fig 7, the biosynthesis pathway of taxadiene introduced to *S*. *cerevisiae* usually consists of ten biochemical reactions, including nine endogenous reactions and one exogenous reaction [38]. Compared with the reported four methods, only PyMiner could completely identify the taxadiene biosynthesis pathway that composed of exact ten reactions in Fig 7. MRE method retrieved two biosynthetic pathways (row 15 in Table 3) that are essentially consistent with Fig 7 but with incomplete information, which means that the lengths of these two pathways are 7 and 8 (instead of 10). In detail, compared with PyMiner, the first pathway (ranked 3rd) retrieved by MRE lacked three biochemical reactions catalyzed by enzyme ERG10 and enzyme ERG20, and the second pathway (ranked 45th) lacked two biochemical reactions catalyzed by enzyme ERG20.

Based on single-step atom mapping, PyMiner exhibited a possible atom transfer route of atoms from substrate acetyl coenzyme A to product taxadiene (Fig 7), and at the same time calculated the key atom utilization of acetyl coenzyme A and key atom conservation of taxadiene to be 0.67 and 0.1 respectively. However, PyMiner only traces the transfer route of atoms in a single molecule, therefore, there may be slight differences between the calculated atom utilization of the initial substrate, the atom conservation of the target product and their corresponding true values. In this example, the theoretical true value of the key atom conservation in this taxadiene synthesis pathway is 1.0, that is, all atoms in taxadiene come from the initial substrate acetyl coenzyme A.

Additionally, PyMiner also identified two other taxadiene synthesis pathways (S3 Table). The main differences of the first pathway (ranked second) from the synthesis pathway shown in Fig 7 are that the biochemical reactions for mevalonate synthesis have changed from an endogenous reaction to an exogenous reaction (S7 Fig), meanwhile the involved cofactors have changed from NADPH to NADH. In addition, in order to maintain a favorable redox state, the NADH-dependent HMG-CoA reductase from *Delftia acidovorans* was introduced into *E*. *coli* to increase the production of amorpha-4,11-diene [44]; the NADH-dependent HMG-CoA reductase from *Bordetella petrii* was introduced into *Yarrowia lipolytica* to increase the production of α‑farnesene [45]; and the NADH-dependent HMG-CoA reductase from *Silicibacter pomeroyi* was introduced into *S*. *cerevisiae* to overproduce squalene [46]. Therefore, it is probably easy to think of constructing this synthetic pathway in *S*. *cerevisiae* may improve the yield of taxadiene. The second pathway (ranked fifth) employs two exogenous enzymes mvaD and ipkA, instead of two endogenous enzymes ERG8 and ERG19, to catalyze the synthesis of isopentenyl diphosphate (S8 Fig). Therein, (R)-5-phosphomevalonate and (R)-mevalonate diphosphate were conferred serious toxicity within mitochondria of *S*. *cerevisiae* [47]. Therefore, the new pathway through intermediate isopentenyl phosphate (instead of (R)-mevalonate diphosphate) provides a new choice for the construction of mevalonate pathway in mitochondria.

## Discussion

In this paper, we propose a novel approach PyMiner (*https*:*//github*.*com/CC-SXF/PyMiner*) as an effective tool for metabolic pathway design. PyMiner integrates biochemical reaction data from KEGG, Rhea, and MetaCyc to construct an EMN based on the uniform similarity between substrate and product, which can meet the requirements of a variety of pathway search, including the metabolic pathways from a given initial substrate to a target product (S9 Fig), and the exogenous pathways of a specific chassis microorganism with a given target product (S2 Fig). Furthermore, PyMiner could not only search for pathways within a given length (S10 Fig), but also search for pathways with a specific length (S3 Fig) or the shortest length (S4 Fig). The user guides of PyMiner are summarized in S1 File.

PyMiner tries to identify biologically feasible metabolic pathways from multiple dimensions. At the first stage of the EMN construction, PyMiner improves the atom utilization and conservation of single-step reactions by generating the main substrate-product pairs of all biochemical reactions. Compared with GMN, a significant decrease level in the number of substrate-product pairs is found, namely 81.62% on average. At the second stage of metabolic pathway search, the LTIOD-based CSS applied in PyMiner can effectively shorten the search time with an average accuracy of 90.6%. At the third stage of metabolic pathway evaluation, PyMiner excludes the metabolic pathways that have no atom transfer by tracing the atom transfer route, and then grants high priority to the metabolic pathway with high atom utilization and high atom conservation. In this process, PyMiner also preferentially selects metabolic pathways with short exogenous and endogenous steps, and as a result, the operation steps of gene manipulation are reduced, and the metabolic burden of a chassis microorganism is decreased. By calculating the main metabolic fluxes of candidate pathways, PyMiner further distinguishes the subtle differences among diverse metabolic pathways, and screens out the pathways with larger synthesis rate of target product.

Compared with state-of-the-art methods on pathway search, PyMiner shows outstanding advantages, and performs equally or better on 95% of representative metabolic pathways. It also demonstrates superiority on the validation sets and the databases employed by the compared methods. Additionally, PyMiner is more prominent in showing the complete details of one metabolic pathway, for instance, the extension of carbon chain.

A smaller value of similarity difference threshold (e.g. ε = 0.1) is helpful to render higher priority to metabolic pathways with higher substrate-atom utilization and higher product-atom conservation, but it may over-constrain the search space of metabolic pathways and lead to fewer results, or no results. For example, we can relax the value of ε to 0.2 and search the metabolic pathways from pyruvate to isopropanol (S3 Table). In addition, the unavailable GSMMs of some chassis microorganisms (such as *Yarrowia lipolytica*) from BIGG restricts the application scenarios of PyMiner. Considering that the computational complexity of the atom transfer route (which is a subgraph matching problem) and the main metabolic flux is relatively high, the evaluation time of metabolic pathway is often much longer than its search time, and shows a positive correlation with the number and length of the retrieved pathways. Therefore, it is necessary to develop intelligent methods to shorten the evaluation time in the future. However, if we don’t have any prior knowledge on the metabolic pathways (e.g. length and number), we can first have a glance at the results returned by PyMiner without evaluation on ‘atom transfer route’ and ‘main metabolic flux’. And in some scenarios, no pathways will be retrieved if CSS is not used (such as the 10 steps pathways from acetyl-CoA to taxadiene as shown in S12 Fig). In addition, since our method only traces the transfer route of atoms in a single molecule, it may result in inevitable differences between the calculated substrate-atom utilization, product-atom conservation and their true values.

Currently, PyMiner mainly employs the length of metabolic pathway, atom utilization and conservation, and main metabolic flux to grant priority to biologically feasible metabolic pathways. We believe that by further integrating the toxicity of metabolites, competitive endogenous pathways and other related information, PyMiner will be able to investigate the candidate metabolic pathways from a broader perspective and give more reasonable suggestions on pathway design.

## Supporting information

S1 Fig The imbalance of metabolic network. (A) The reaction-number imbalance of metabolic network structure. The imbalance is mainly reflected in the complexity of primary metabolism and the relative simplicity of secondary metabolism, that is, the average reaction number of secondary and non-secondary metabolites are 3.2 and 16, respectively. All secondary or non-secondary metabolites are derived from the validation dataset of 2812 metabolic pathways established in PyMiner, and the classification standard is the occurrence or non-occurrence of ‘Secondary Metabolite’ in their class descriptions. In total, 741 secondary metabolites and 692 non-secondary metabolites are retrieved. P value is calculated based on two-sample t-test. (B) The difference of search time related to the imbalance of metabolic network structure. Using the information from the 741 secondary metabolites, the 692 non-secondary metabolites and the 2812 metabolic pathways, we identified 490 (out of 2812) pathways, of which the initial substrates belong to non-secondary metabolites (or secondary metabolites), and the target products belong to secondary metabolites (or non-secondary metabolites). Metabolic pathway searches (including forward search strategy and reverse search strategy) were performed on these 490 pathways, and the search times were recorded. The statistical information of the search times from non-secondary (primary) metabolites to secondary metabolites and the search times from secondary metabolites to non-secondary (primary) metabolites is shown in panel B. P value is calculated based on two sample t-test.
(TIF)

S2 Fig The exogenous pathways of Escherichia coli retrieved by PyMiner for the biosynthesis of resveratrol. It demonstrates an application case for the exogenous pathway design of a specific chassis microorganism just given target product. Key inputs applied in this example were: *Sources*, {}; *Target*, Met001306-m; Host Organism, eco; *Database*, MetaCyc; and *Maximum Length*, 4. Additionally, the default value of other parameters was employed.
(TIF)

S3 Fig The biosynthesis pathway of xylitol from D-xylose extracted by PyMiner. This demo illustrates an application case for pathway design with a specific length. Key inputs employed in this case were: *Sources*, {"C00181"}; *Target*, C00379; *Host Organism*, eco; *Database*, KEGG; *Maximum Length*, 2; and *Total*, unchecked. Moreover, the default value of other inputs was used.
(TIF)

S4 Fig The shortest biosynthetic pathway of artemisinate from acetyl-CoA identified by PyMiner. This case study displays an application case for pathway design with shortest length. Key inputs adopted in this example were: *Sources*, {"Met000025-m"}; *Target*, Met002678-m; *Host Organism*, sce; *Database*, KndPad; *Maximum Length*, 16; and *Shortest*, checked. Additionally, the default value of other inputs was employed.
(TIF)

S5 FigCalculation method of the main metabolic flux of one representative metabolic pathway.(A) A representative metabolic pathway from initial substrate to target product. The blue dashed arrow means multi-step reactions. *C*_*i*_, *C*_*j*_ *and C*_*k*_ are given as examples of the main intermediate metabolites or target product of one metabolic pathway. *W*_*i*_, *W*_*j*_ and *W*_*k*_ are the numbers of reactions that metabolites *C*_*i*_, *C*_*j*_ and *C*_*k*_ participate in. *v*_*i*1_, *v*_*j*1_ and *v*_*k*1_ are main metabolic fluxes, and $v_{i2},\cdots ,v_{iW_{i}},v_{j2},\cdots ,v_{jW_{j}},v_{k2},\cdots ,v_{kW_{k}}$ are branching metabolic fluxes. (B) The constraints (such as $v_{i1}\geq v_{i2},v_{i1}\geq v_{i3},\cdots ,v_{i1}\geq v_{iW_{i}}$) that the main metabolic flux must meet. These constraints are very important for distinguishing metabolic pathways of which the endogenous parts are different, but the exogenous parts are the same. After integrating the same exogenous part (exogenous reactions) into a GSMM, the new GSMMs and the objective functions corresponding to these pathways are identical. Therefore, the metabolic fluxes (that is, synthesis rates) corresponding to the target product are the same. (C) Equivalent constraints (such as $v_{t2}\geq 0,v_{t3}\geq 0,\cdots ,v_{tW_{i}}\geq 0$) to be satisfied by the main metabolic flux after appending additional pseudo metabolites ($e.g.M_{t2},M_{t3},\cdots ,M_{tW_{i}}$) and pseudo reactions (*e.g.* $R_{t2},R_{t3},\cdots ,R_{tW_{i}}$) by using COBRApy.(TIF)

S6 Fig The statistical information of substrate-product pairs. *Inf* means the total numbers of substrate-product pairs without removing any general cofactors (GC). After removing GC, all substrate-product pairs accompanied by carbon atom transfer were constructed at *ε* = 1.0. Lower value in ε indicates a stricter standard and leads to decreases in number of substrate-product pairs. If *ε* decreases to 0.1, compared to *ε* = 1.0, the numbers of substrate-product pairs are shown to decrease by 15.27% (KEGG), 19.40% (MetaCyc), and 19.84% (KndPad), respectively. However, compared to *Inf*, more significant decrease levels are found at *ε* = 0.1, namely, 79.63% (KEGG), 82.94% (MetaCyc) and 82.28% (KndPad). Therefore, redundant information is eliminated, and the numbers of substrate-product pairs are shown to decrease by 81.62% on average.
(TIF)

S7 Fig The second biosynthesis pathway of taxadiene in *S*. *cerevisiae* retrieved by PyMiner. Instead of the endogenous HMGR, exogenous enzyme mvaA catalyzes the synthesis of (R)-Mevalonate with the participation of NADH. The green arrows indicate endogenous steps while the blue arrows denote exogenous steps. The green circles highlight the atom transfer route from acetyl-CoA to taxadiene. The abbreviations are: ERG10, acetyl-CoA acetyltransferase; HMGS, HMG-CoA synthase; mvaA, hydroxymethylglutaryl-CoA reductase; ERG12, mevalonate kinase; ERG8, phosphomevalonate kinase; ERG19, mevalonate diphosphate decarboxylase; ERG20, farnesyl diphosphate synthase; and BTS1, geranylgeranyl diphosphate synthase.
(TIF)

S8 Fig The fifth biosynthesis pathway of taxadiene in *S*. *cerevisiae* identified by PyMiner. Instead of two endogenous reactions catalyzed by ERG8 and ERG19, two exogenous enzymes namely mvaD and ipkA catalyze the synthesis of isopentenyl diphosphate. The green arrows indicate endogenous steps while the blue arrows denote exogenous steps. The green circles highlight the atom transfer route from acetyl-CoA to taxadiene. The abbreviations are: ERG10, acetyl-CoA acetyltransferase; HMGS, HMG-CoA synthase; HMGR, HMG-CoA reductase; ERG12, mevalonate kinase; mvaD, phosphomevalonate decarboxylase; ipkA, isopentenyl phosphate kinase; ERG20, farnesyl diphosphate synthase; and BTS1, geranylgeranyl diphosphate synthase.
(TIF)

S9 Fig The biosynthetic pathways of ethylene glycol from aldehydo-D-xylose identified by PyMiner. This example shows an application case for pathway design given initial substrates and target product. Key inputs used here were: *Sources*, {"Met005802-m"}; *Target*, Met001390-m; *Host Organism*, eco; *Database*, MetaCyc; *Maximum Length*, 4; and *Infeasibility*, checked. Furthermore, the default value of other inputs was adopted. Red arrow represents an infeasible reaction, which implies the necessity of adding aldehydo-D-xylose to the culture medium.
(TIF)

S10 Fig A snapshot of PyMiner. The demo example shows the candidate biosynthetic pathways of resveratrol (C03582) retrieved by PyMiner from start substrates L-phenylalanine (C00079) and L-tyrosine (C00082). Key inputs entered into PyMiner were: *Sources*, {"C00079", "C00082"}; *Target*, C03582; *Host Organism*, eco; *Database*, KEGG; and *Maximum Length*, 4. In addition, the default value of other parameters was used.
(TIF)

S11 Fig A snapshot of *Tips* in PyMiner. Helpful prompt messages are real-time displayed through all the cycle of pathway design, including the input period, the search period and the evaluation period. In this demo, 3 pathways in total were identified, as displayed in *Tips*.
(TIF)

S12 Fig The importance of conditional search strategy (CSS). (A) Forward search and breadth first search (BFS); (B) Forward search and depth first search (DFS); (C) The CSS based on LTIOD and BFS. The three panels are snapshots of Tips in PyMiner.
(TIF)

S1 File The application of PyMiner. (PDF)

S2 File The pseudo codes of PyMiner. (PDF)

S1 Table The cleaned reactions and compounds of KndPad and the validation dataset of 2812 linear metabolic pathways. (XLSX)

S2 Table The general cofactors of KEGG, MetaCyc and KndPad. (XLSX)

S3 Table The 20 experimentally verified pathways applied to compare with other 4 methods. (XLSX)

S4 Table The validation sets employed by RouteSearch, MRE, EcoSynther and PATH^cre8^. (XLSX)
