# Supplementary figures and images for: PyMiner: A method for metabolic pathway design based on the uniform similarity of substrate-product pairs and conditional search
Source: PLoS One. 2022 Apr 11;17(4):e0266783. doi: 10.1371/journal.pone.0266783 (PMC9000129; doi:10.1371/journal.pone.0266783)

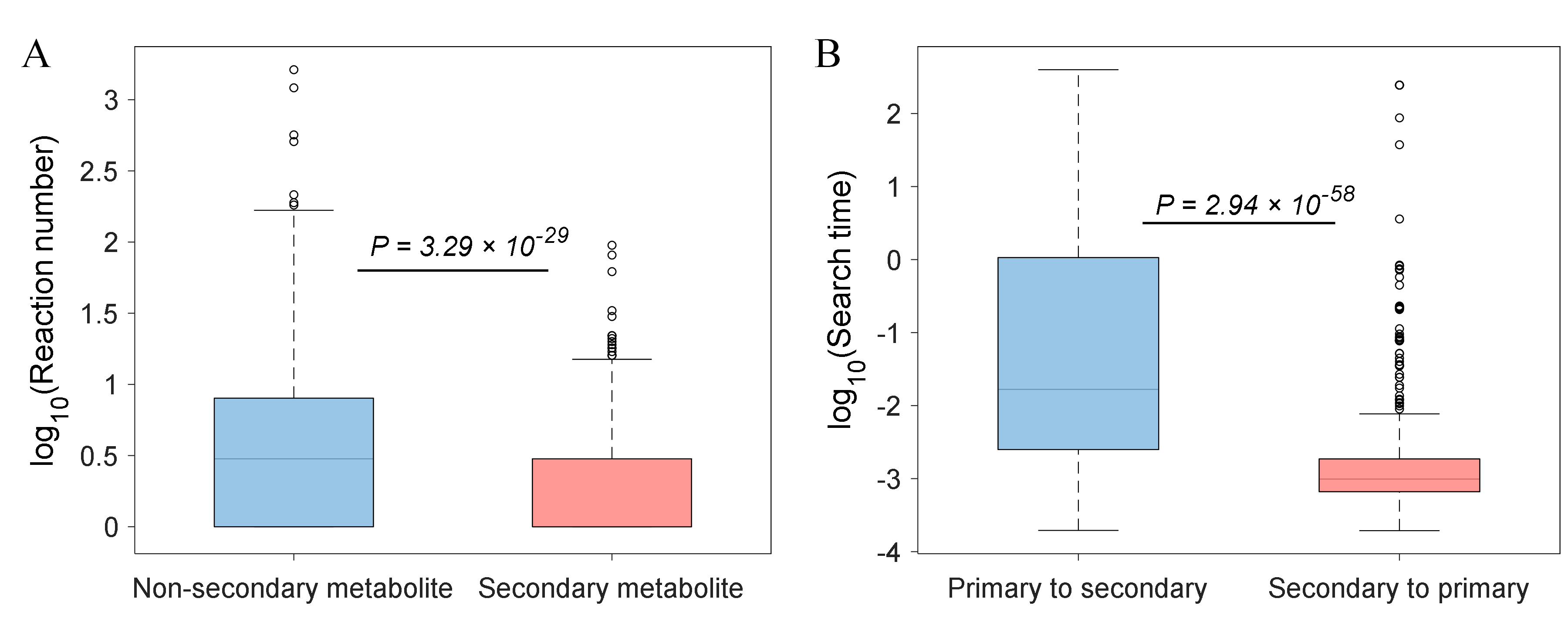

Supplement: S1 Fig — (A) The reaction-number imbalance of metabolic network structure. The imbalance is mainly reflected in the complexity of primary metabolism and the relative simplicity of secondary metabolism, that is, the average reaction number of secondary and non-secondary metabolites are 3.2 and 16, respectively. All secondary or non-secondary metabolites are derived from the validation dataset of 2812 metabolic pathways established in PyMiner, and the classification standard is the occurrence or non-occurrence of ‘Secondary Metabolite’ in their class descriptions. In total, 741 secondary metabolites and 692 non-secondary metabolites are retrieved. P value is calculated based on two-sample t-test. (B) The difference of search time related to the imbalance of metabolic network structure. Using the information from the 741 secondary metabolites, the 692 non-secondary metabolites and the 2812 metabolic pathways, we identified 490 (out of 2812) pathways, of which the initial substrates belong to non-secondary metabolites (or secondary metabolites), and the target products belong to secondary metabolites (or non-secondary metabolites). Metabolic pathway searches (including forward search strategy and reverse search strategy) were performed on these 490 pathways, and the search times were recorded. The statistical information of the search times from non-secondary (primary) metabolites to secondary metabolites and the search times from secondary metabolites to non-secondary (primary) metabolites is shown in panel B. P value is calculated based on two sample t-test. (TIF) [file pone.0266783.s001.tif]

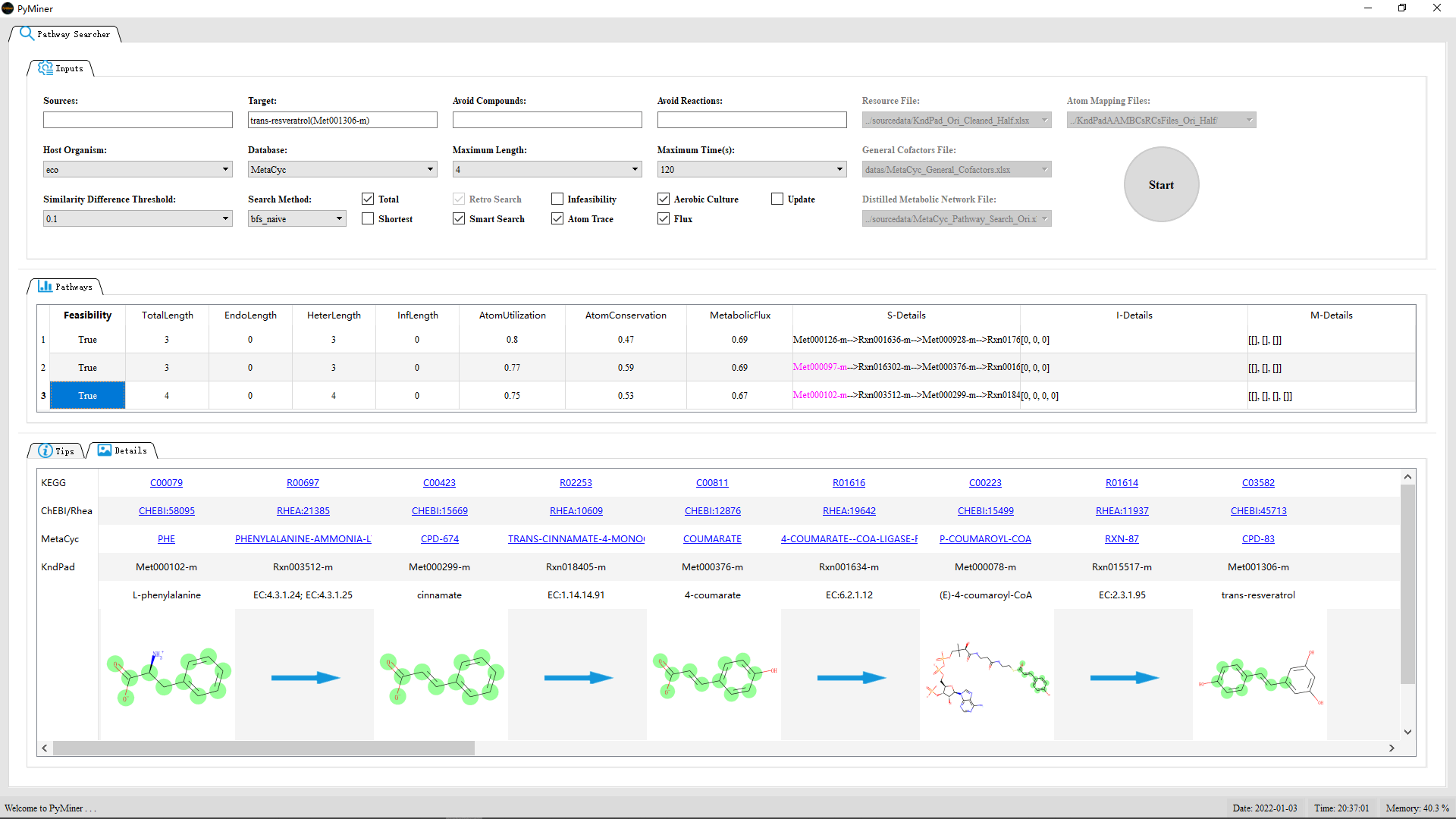

Supplement: S2 Fig — It demonstrates an application case for the exogenous pathway design of a specific chassis microorganism just given target product. Key inputs applied in this example were: Sources, {}; Target, Met001306-m; Host Organism, eco; Database, MetaCyc; and Maximum Length, 4. Additionally, the default value of other parameters was employed. (TIF) [file pone.0266783.s002.tif]

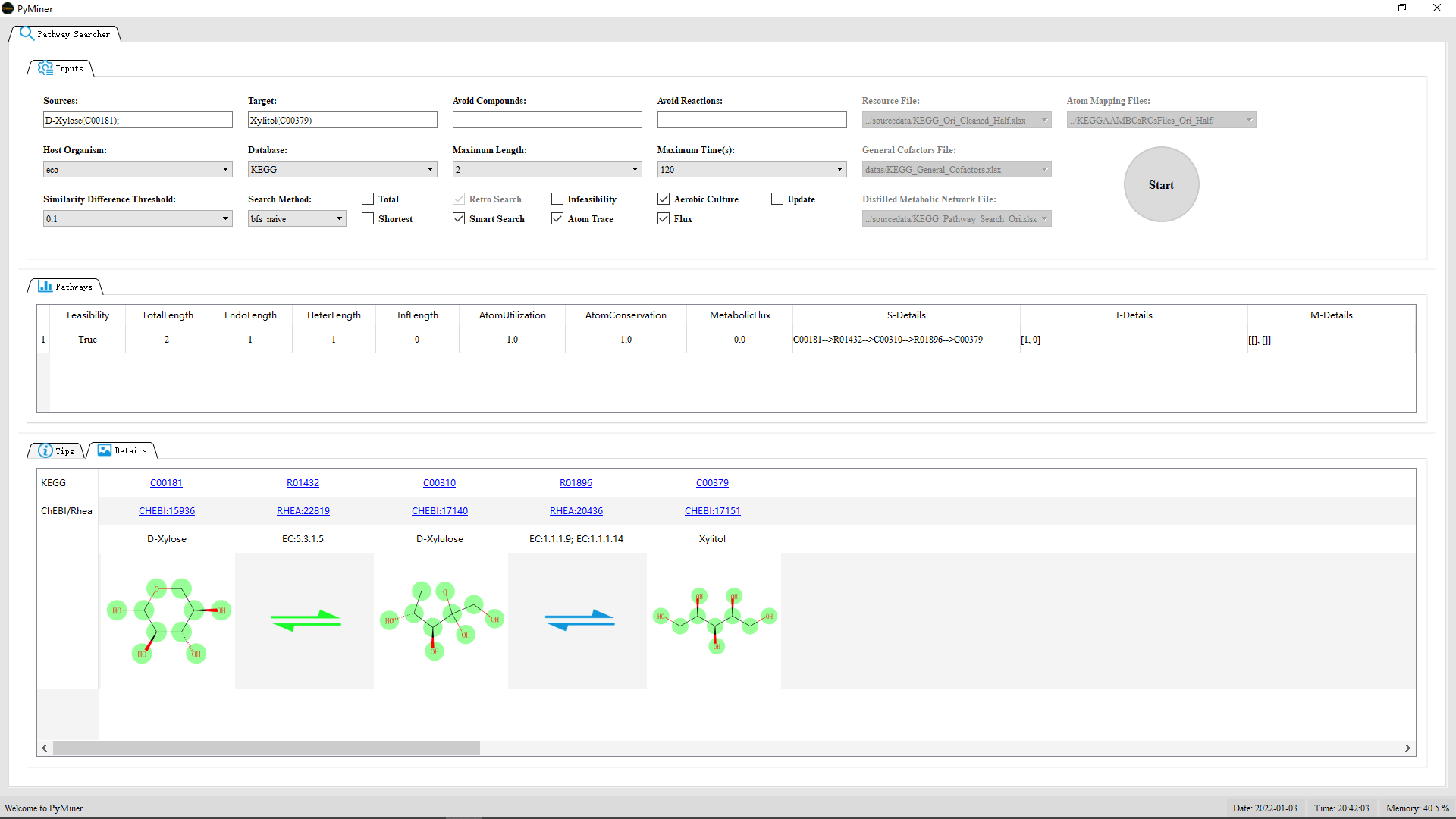

Supplement: S3 Fig — This demo illustrates an application case for pathway design with a specific length. Key inputs employed in this case were: Sources, {"C00181"}; Target, C00379; Host Organism, eco; Database, KEGG; Maximum Length, 2; and Total, unchecked. Moreover, the default value of other inputs was used. (TIF) [file pone.0266783.s003.tif]

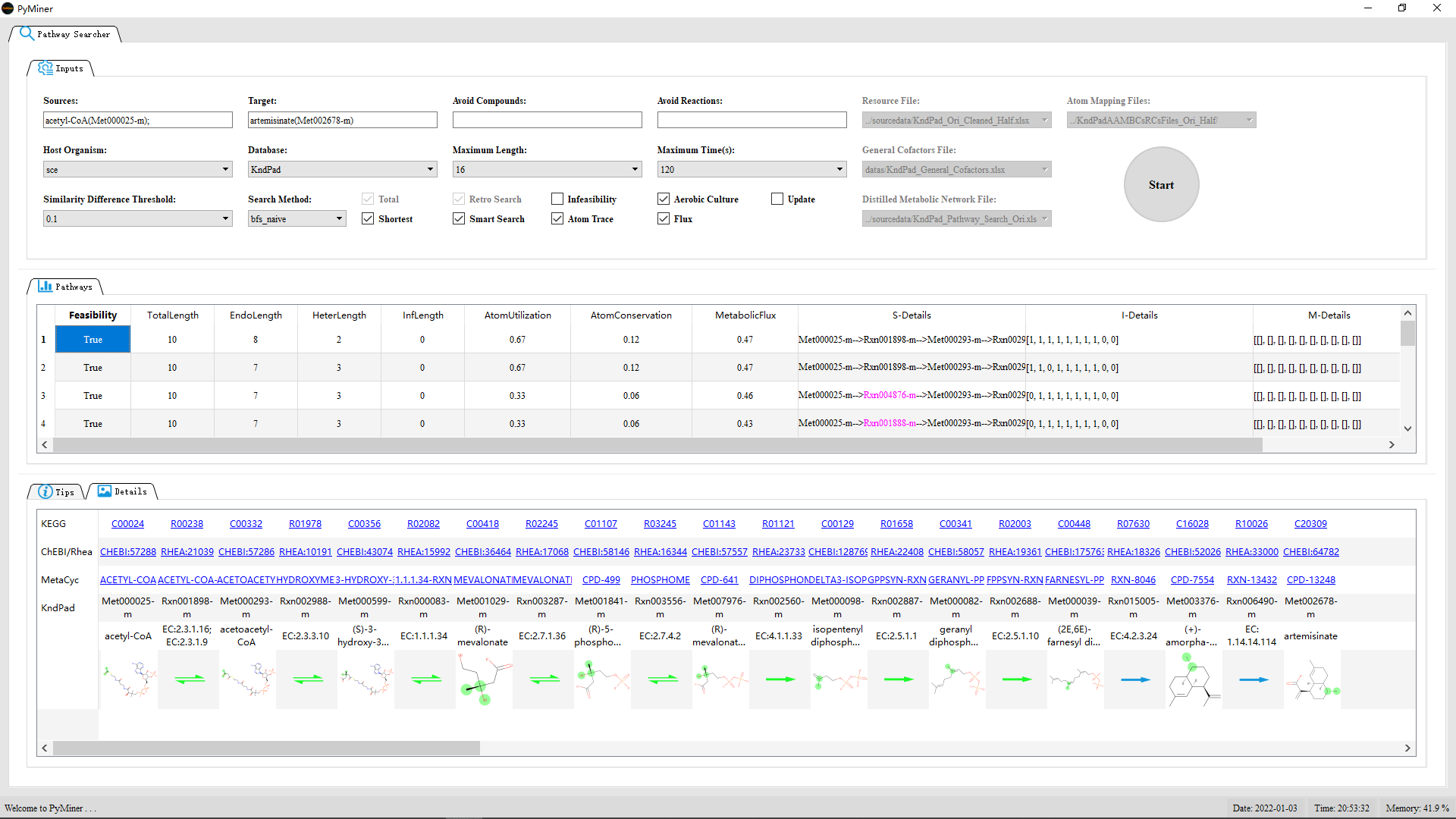

Supplement: S4 Fig — This case study displays an application case for pathway design with shortest length. Key inputs adopted in this example were: Sources, {"Met000025-m"}; Target, Met002678-m; Host Organism, sce; Database, KndPad; Maximum Length, 16; and Shortest, checked. Additionally, the default value of other inputs was employed. (TIF) [file pone.0266783.s004.tif]

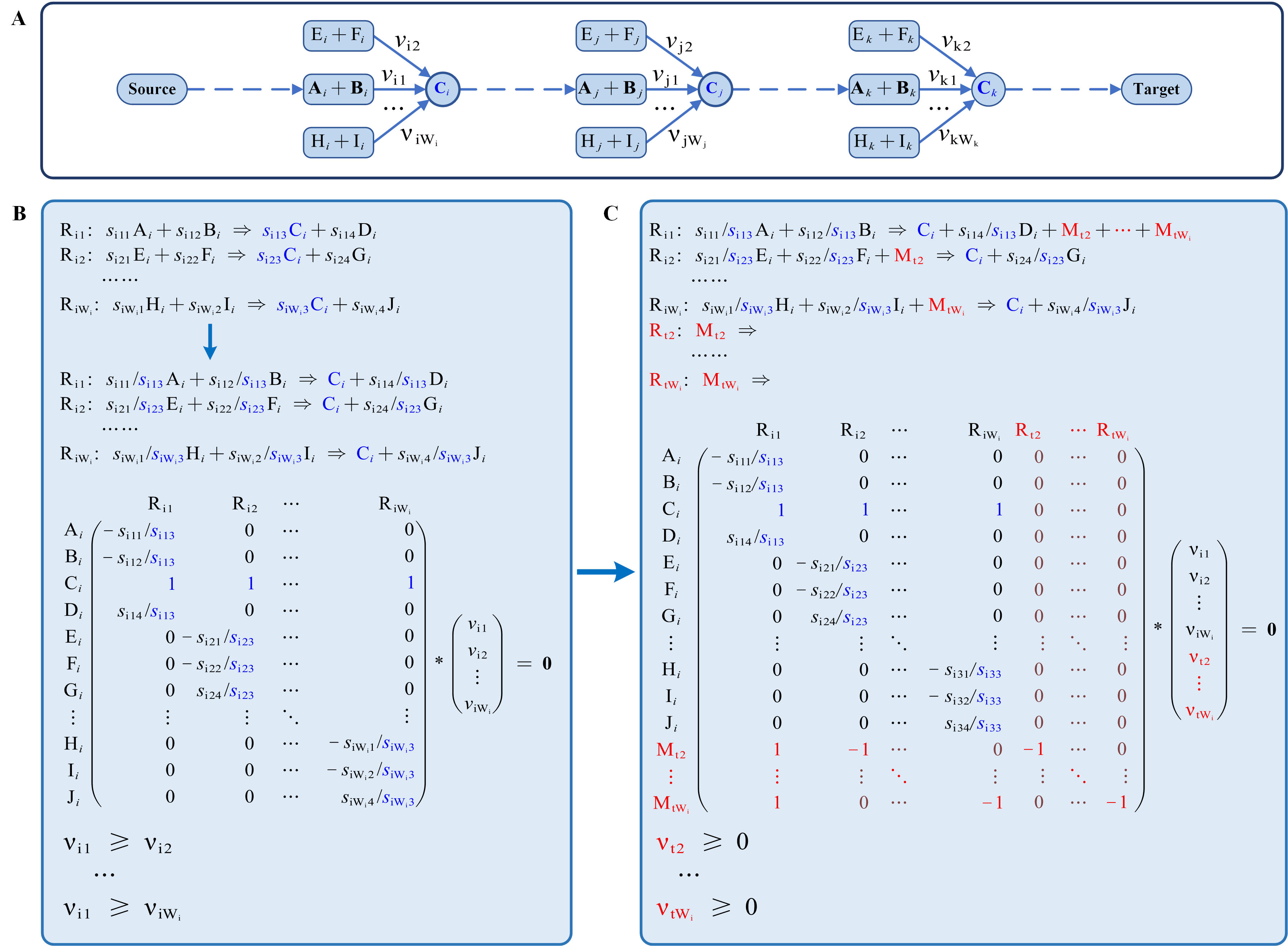

Supplement: S5 Fig — (A) A representative metabolic pathway from initial substrate to target product. The blue dashed arrow means multi-step reactions. Ci, Cj and Ck are given as examples of the main intermediate metabolites or target product of one metabolic pathway. Wi, Wj and Wk are the numbers of reactions that metabolites Ci, Cj and Ck participate in. vi1, vj1 and vk1 are main metabolic fluxes, and vi2,⋯,viWi,vj2,⋯,vjWj,vk2,⋯,vkWk are branching metabolic fluxes. (B) The constraints (such as vi1≥vi2,vi1≥vi3,⋯,vi1≥viWi) that the main metabolic flux must meet. These constraints are very important for distinguishing metabolic pathways of which the endogenous parts are different, but the exogenous parts are the same. After integrating the same exogenous part (exogenous reactions) into a GSMM, the new GSMMs and the objective functions corresponding to these pathways are identical. Therefore, the metabolic fluxes (that is, synthesis rates) corresponding to the target product are the same. (C) Equivalent constraints (such as vt2≥0,vt3≥0,⋯,vtWi≥0) to be satisfied by the main metabolic flux after appending additional pseudo metabolites (e.g.Mt2,Mt3,⋯,MtWi) and pseudo reactions (e.g. Rt2,Rt3,⋯,RtWi) by using COBRApy. (TIF) [file pone.0266783.s005.tif]

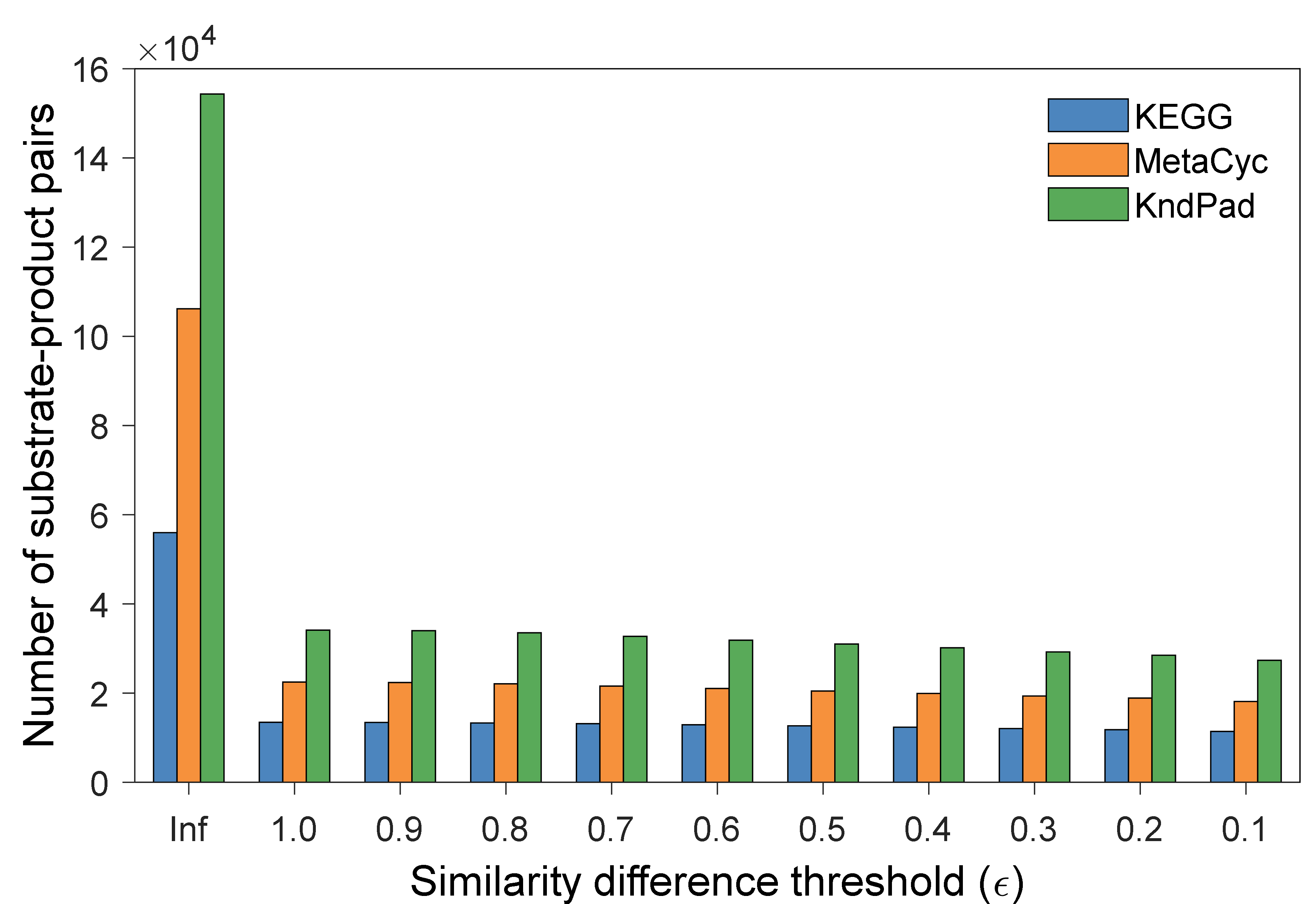

Supplement: S6 Fig — Inf means the total numbers of substrate-product pairs without removing any general cofactors (GC). After removing GC, all substrate-product pairs accompanied by carbon atom transfer were constructed at ε = 1.0. Lower value in ε indicates a stricter standard and leads to decreases in number of substrate-product pairs. If ε decreases to 0.1, compared to ε = 1.0, the numbers of substrate-product pairs are shown to decrease by 15.27% (KEGG), 19.40% (MetaCyc), and 19.84% (KndPad), respectively. However, compared to Inf, more significant decrease levels are found at ε = 0.1, namely, 79.63% (KEGG), 82.94% (MetaCyc) and 82.28% (KndPad). Therefore, redundant information is eliminated, and the numbers of substrate-product pairs are shown to decrease by 81.62% on average. (TIF) [file pone.0266783.s006.tif]

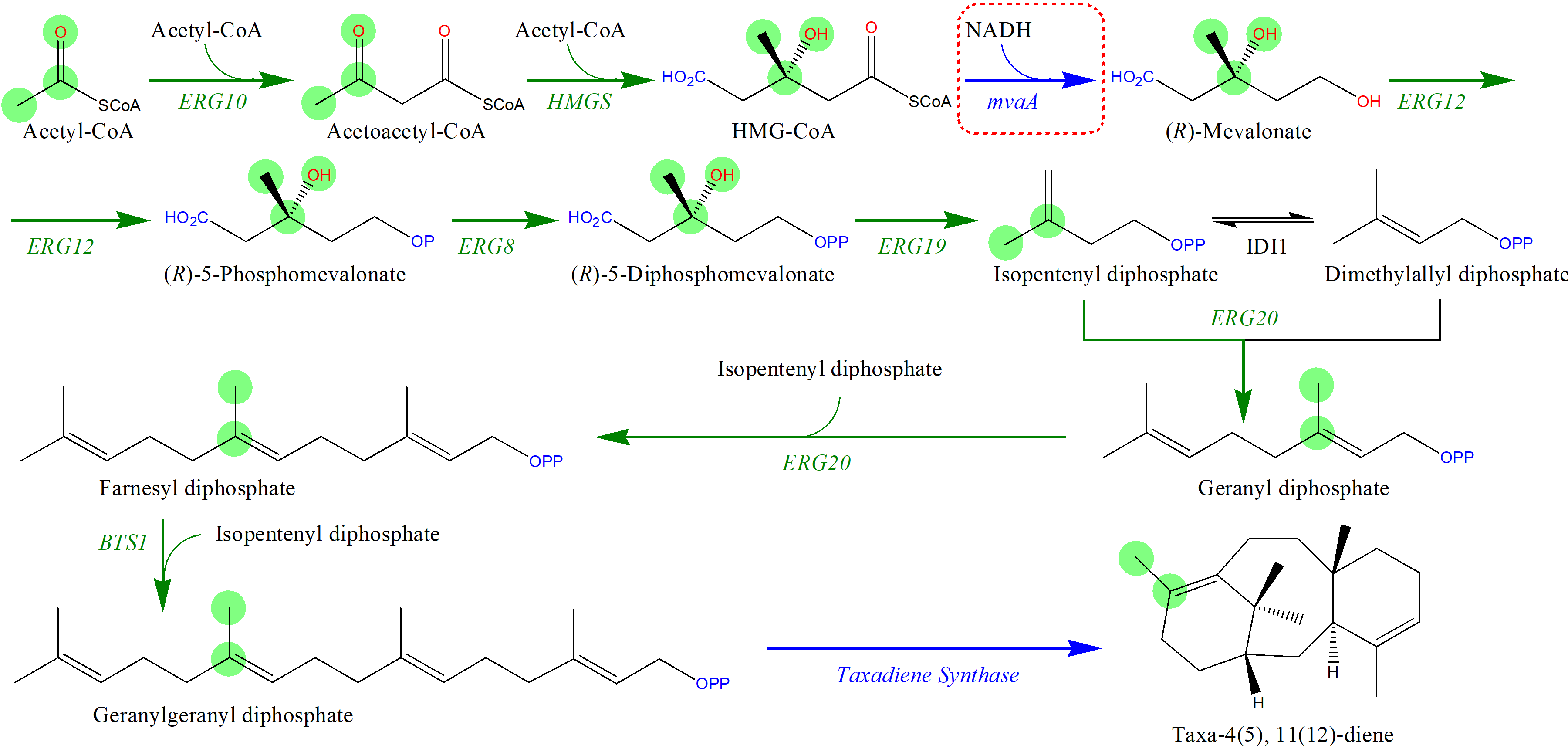

Supplement: S7 Fig — Instead of the endogenous HMGR, exogenous enzyme mvaA catalyzes the synthesis of (R)-Mevalonate with the participation of NADH. The green arrows indicate endogenous steps while the blue arrows denote exogenous steps. The green circles highlight the atom transfer route from acetyl-CoA to taxadiene. The abbreviations are: ERG10, acetyl-CoA acetyltransferase; HMGS, HMG-CoA synthase; mvaA, hydroxymethylglutaryl-CoA reductase; ERG12, mevalonate kinase; ERG8, phosphomevalonate kinase; ERG19, mevalonate diphosphate decarboxylase; ERG20, farnesyl diphosphate synthase; and BTS1, geranylgeranyl diphosphate synthase. (TIF) [file pone.0266783.s007.tif]

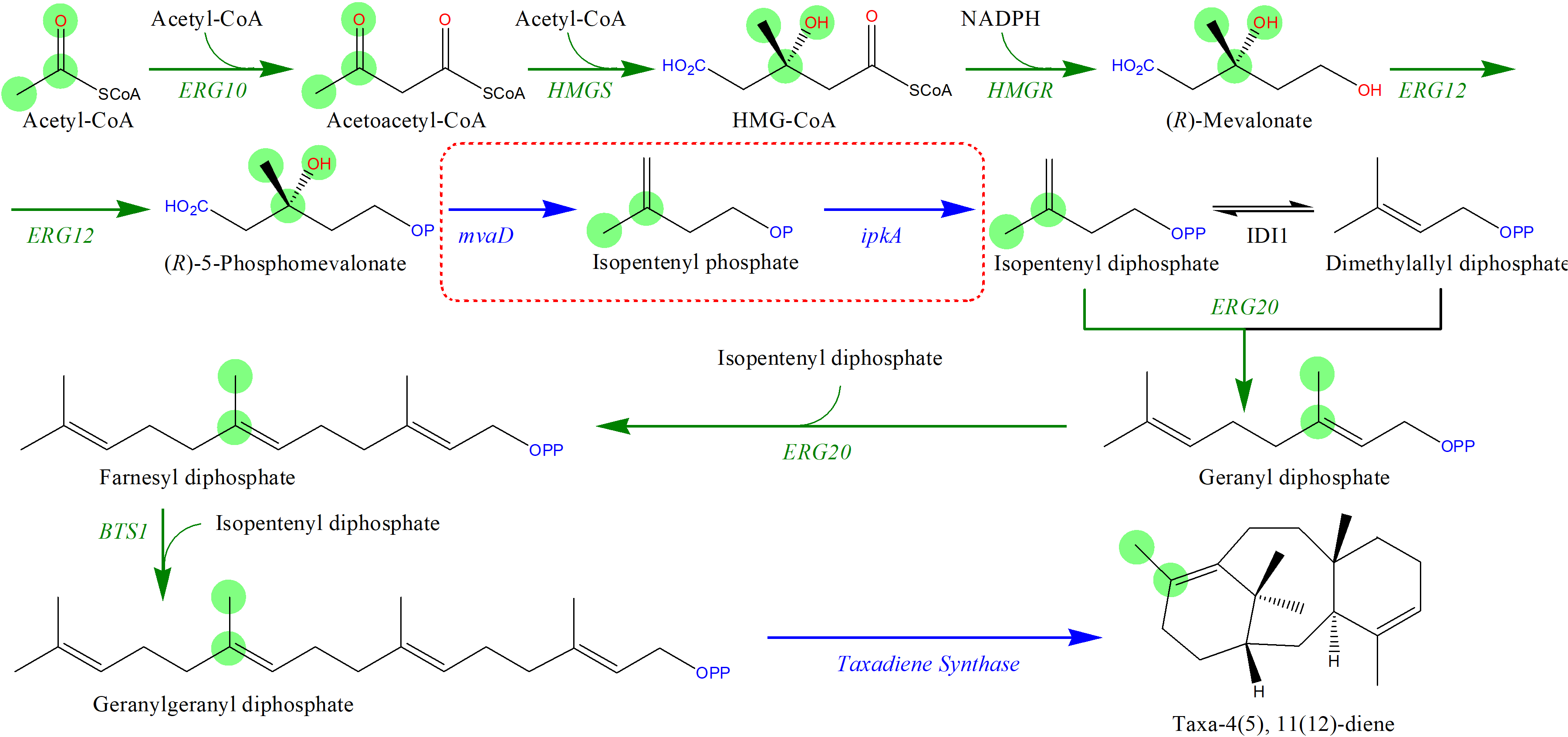

Supplement: S8 Fig — Instead of two endogenous reactions catalyzed by ERG8 and ERG19, two exogenous enzymes namely mvaD and ipkA catalyze the synthesis of isopentenyl diphosphate. The green arrows indicate endogenous steps while the blue arrows denote exogenous steps. The green circles highlight the atom transfer route from acetyl-CoA to taxadiene. The abbreviations are: ERG10, acetyl-CoA acetyltransferase; HMGS, HMG-CoA synthase; HMGR, HMG-CoA reductase; ERG12, mevalonate kinase; mvaD, phosphomevalonate decarboxylase; ipkA, isopentenyl phosphate kinase; ERG20, farnesyl diphosphate synthase; and BTS1, geranylgeranyl diphosphate synthase. (TIF) [file pone.0266783.s008.tif]

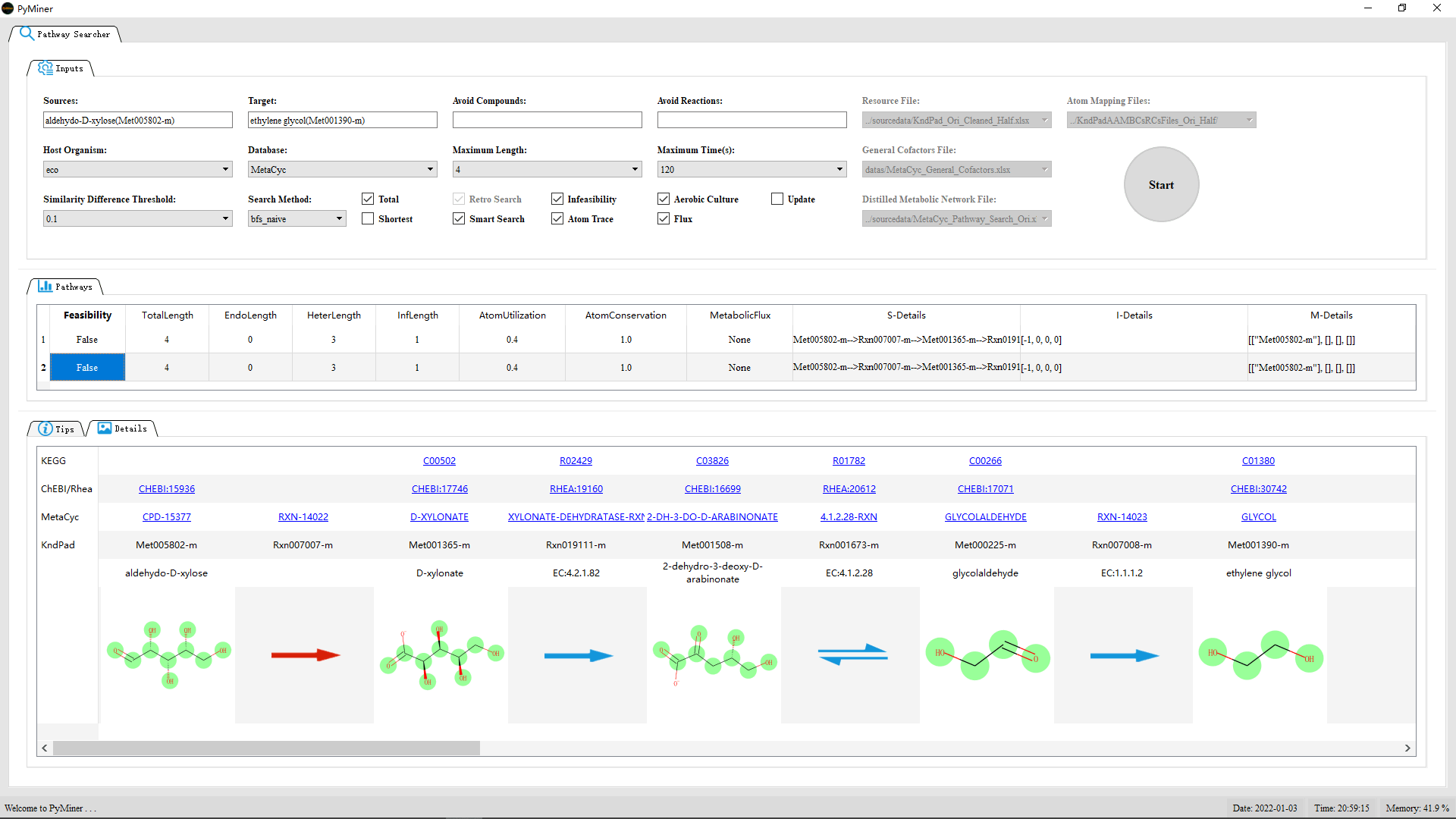

Supplement: S9 Fig — This example shows an application case for pathway design given initial substrates and target product. Key inputs used here were: Sources, {"Met005802-m"}; Target, Met001390-m; Host Organism, eco; Database, MetaCyc; Maximum Length, 4; and Infeasibility, checked. Furthermore, the default value of other inputs was adopted. Red arrow represents an infeasible reaction, which implies the necessity of adding aldehydo-D-xylose to the culture medium. (TIF) [file pone.0266783.s009.tif]

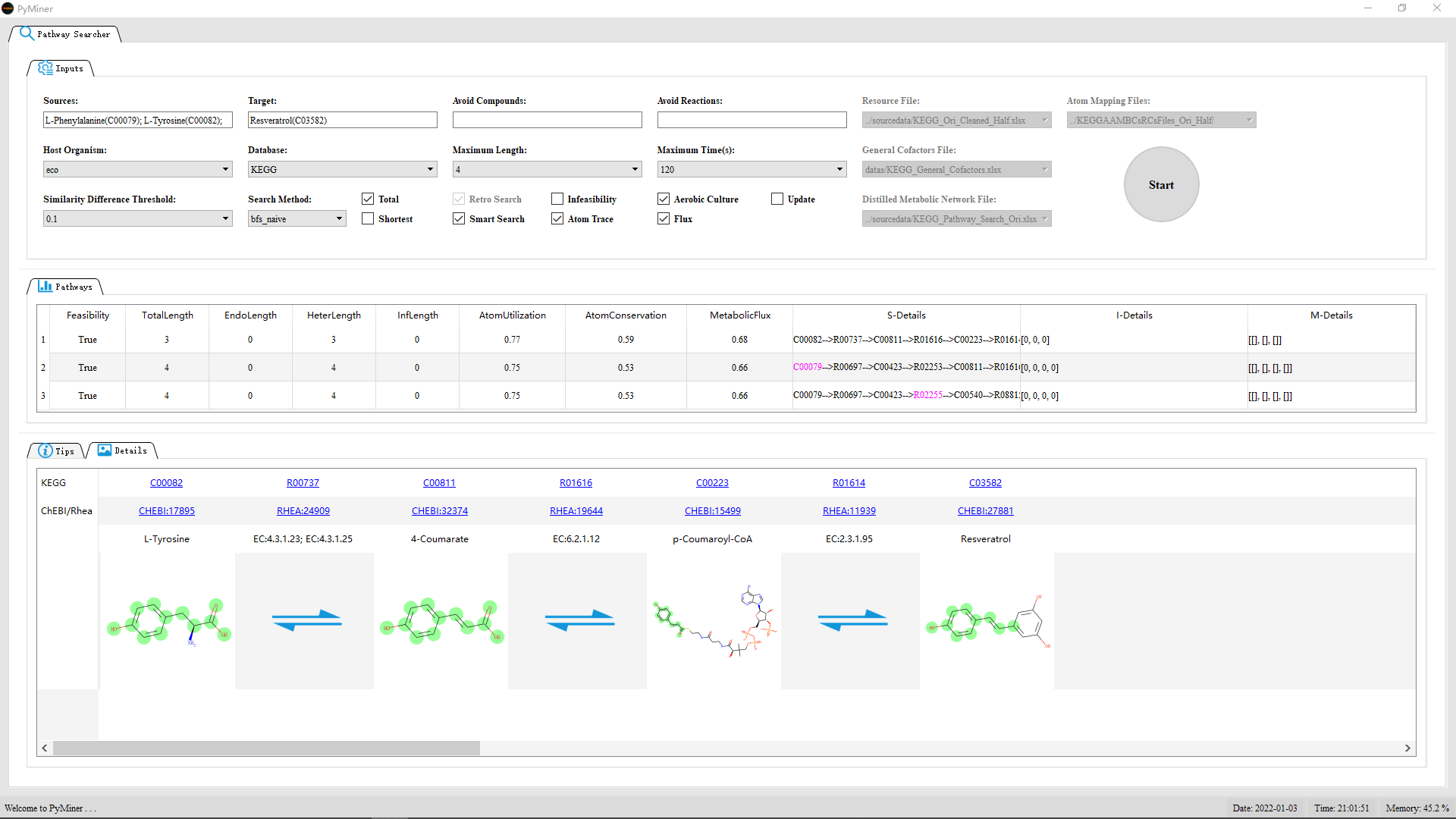

Supplement: S10 Fig — The demo example shows the candidate biosynthetic pathways of resveratrol (C03582) retrieved by PyMiner from start substrates L-phenylalanine (C00079) and L-tyrosine (C00082). Key inputs entered into PyMiner were: Sources, {"C00079", "C00082"}; Target, C03582; Host Organism, eco; Database, KEGG; and Maximum Length, 4. In addition, the default value of other parameters was used. (TIF) [file pone.0266783.s010.tif]

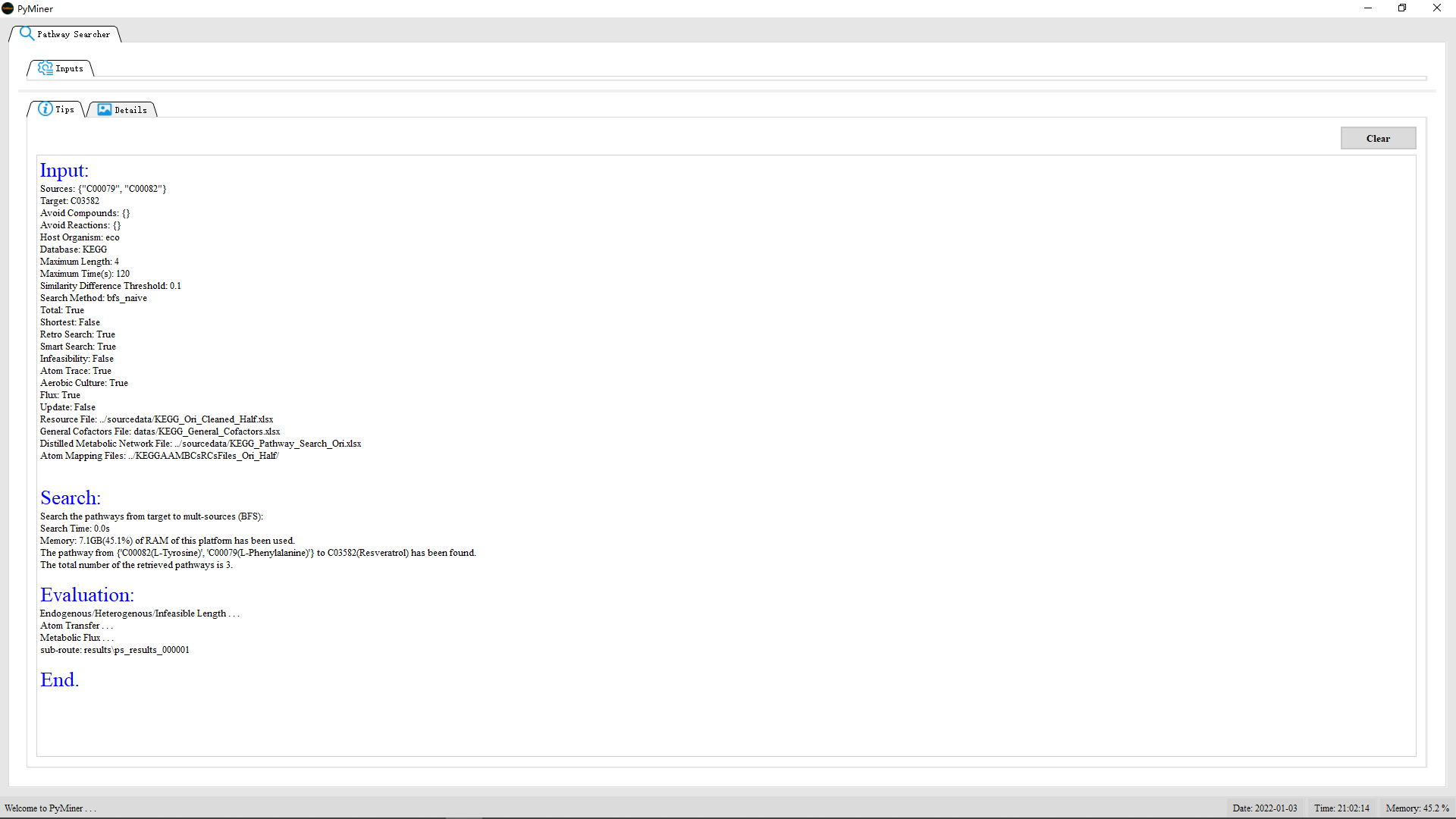

Supplement: S11 Fig — Helpful prompt messages are real-time displayed through all the cycle of pathway design, including the input period, the search period and the evaluation period. In this demo, 3 pathways in total were identified, as displayed in Tips. (TIF) [file pone.0266783.s011.tif]

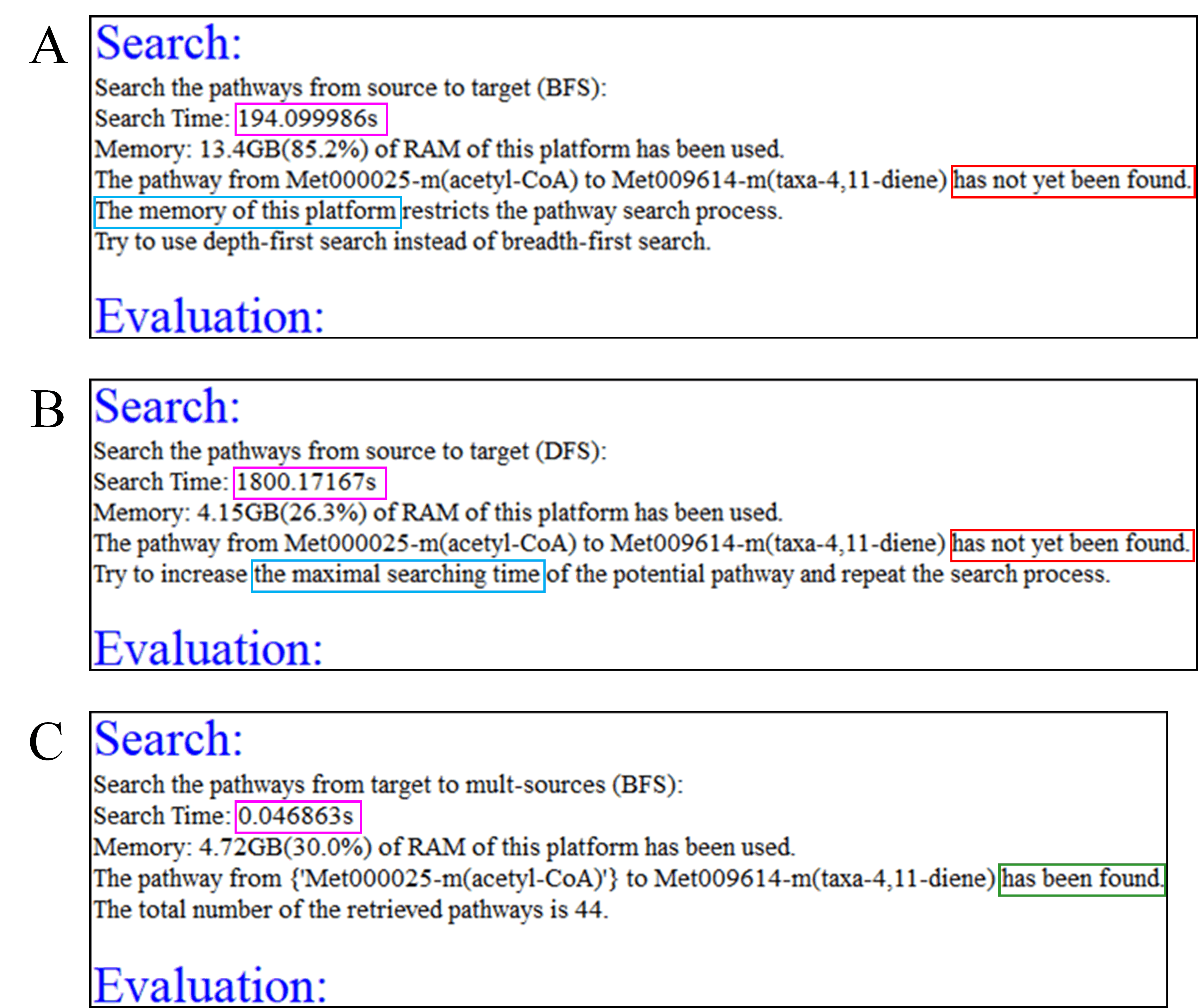

Supplement: S12 Fig — (A) Forward search and breadth first search (BFS); (B) Forward search and depth first search (DFS); (C) The CSS based on LTIOD and BFS. The three panels are snapshots of Tips in PyMiner. (TIF) [file pone.0266783.s012.tif]
