# Supplementary material for: PyMiner: A method for metabolic pathway design based on the uniform similarity of substrate-product pairs and conditional search
Source: PLoS One. 2022 Apr 11;17(4):e0266783. doi: 10.1371/journal.pone.0266783 (PMC9000129; doi:10.1371/journal.pone.0266783)
Supplement: S1 File — (PDF) [file pone.0266783.s013.pdf]

Supplementary information for PyMiner: A Method for Metabolic Pathway Design Based on the Uniform Similarity of Substrate-Product Pairs and Conditional Search

Xinfang Song¶, Mingyu Dong¶ and Min Liu*

Department of Automation, Tsinghua University, Beijing 100084, China

* Corresponding author:

Email: lium@tsinghua.edu.cn (ML)

¶These authors contributed equally to this work.

The application of PyMiner

PyMiner is a practical and effective tool for metabolic pathway design that can meet a variety of pathway design requirements, including the pathways from initial substrates (e.g., L-phenylalanine and L-tyrosine) to target product (e.g., resveratrol) (S10 Fig), and the exogenous pathways of one specific chassis microorganism (e.g., *Escherichia coli*) just given the target product (S2 Fig). In addition, PyMiner can search for metabolic pathways within a given length (S9 Fig), metabolic pathways with a specific length (S3 Fig), and the shortest metabolic pathways (S4 Fig). Please perform the following steps for the installation and operation of PyMiner.

Step 1 File decompression

Before configuring the operating environment of PyMiner, some packed files should be unpacked to their corresponding paths. In details, temporary files 'KEGG_Debug.tar.gz', 'KEGG_Dmn.tar.gz', 'KndPad_Debug.tar.gz', 'KndPad_Dmn.tar.gz', 'MetaCyc_Debug.tar.gz' and 'KndPad_Dmn.tar.gz' in './temp/' should be unpacked; atom-atom mapping files within compressed file 'KEGGAAMBCsRCsFiles_Ori_Half.tar.gz' should be extracted to '../KEGGAAMBCsRCsFiles_Ori_Half/'; atom-atom mapping files stored in the following three compressed files 'KndPadAAMBCsRCsFiles_Ori_Half_part_1.tar.gz', 'KndPadAAMBCsRCsFiles_Ori_Half_part_2.tar.gz' and 'KndPadAAMBCsRCsFiles_Ori_Half_part_3.tar.gz' should be sequentially unpacked to the destination path '../ KndPadAAMBCsRCsFiles_Ori_Half/'. For convenience, an unzip script (unpack_resource_files.py) is provided in source code.

Step 2 Operating environment configuration

Anaconda environment

Windows:

https://repo.anaconda.com/archive/Anaconda3-2019.07-Windows-x86_64.exe

—> Add Anaconda to the system PATH environment variable.

MacOS:

https://repo.anaconda.com/archive/Anaconda3-2019.07-MacOSX-x86_64.pkg

PyQt5, RDKit (https://www.rdkit.org/) and COBRApy [1] were used to build the metabolic pathway design tool PyMiner. To apply PyMiner in metabolic pathway design, please install the following tools:

1) PyQt5 (satisfied after installing Anaconda3)

https://pypi.org/project/PyQt5/5.9.2/

pip install PyQt5==5.9.2

2) numpy (so as to be compatible with COBRApy)

https://numpy.org/install/

pip install numpy==1.17.3

3) RDKit

https://anaconda.org/rdkit/rdkit

conda install -c rdkit rdkit=2020.03.2

4) COBRApy

https://pypi.org/project/cobra/0.22.0/

pip install cobra==0.22.0

Step 3 An overview of the PyMiner

As shown in S10 Fig, there are 4 primary components in PyMiner, encompassing *Inputs*, *Pathways*, *Tips* and *Details*. *Inputs* consists of modifiable and default parameters used for pathway design. *Pathways* lists all the retrieved biosynthesis routes of the target product as well as their basic properties, and automatically updates once the pathway search has been finished. *Tips* provides some helpful prompt messages through all the cycle of

pathway design, including the input period, the search period and the evaluation period (S11 Fig). *Details* demonstrates the detailed information of the pathway that selected from *Pathways*.

3.1 **Inputs**

Sources gives the initial substrates whose valid inputs format are compound IDs (e.g., C00079 and C00082). According to the requirement of the pathway design, one can enter more than one substrate in a multi-input mode. If users are exclusively interested in the heterogenous pathways of one specific chassis microorganism, then an empty value can be set for this input field. In addition, candidate compounds are automatically completed below the *Sources* box while entering the starting characters of one new substrate. What's more, considering the demand of multi-input field, the Enter key must be used to confirm a new substrate or completely clear this input field.

Target gives the target product, and also the valid input format is compound ID (e.g., C03582). Potential compounds are automatically listed below the *Target* box while entering the starting characters of a new target product. Here, only single input is supported.

Avoid Compounds gives a set of molecules that will be excluded from the extracted metabolic network (EMN), for example, acetyl-CoA (C00024) can be entered in this set if we want to retrieve the methyl-D-erythritol-4-phosphate (MEP) pathway. Furthermore, the multi-input mode of *Avoid Compounds* is identical to *Sources*.

Avoid Reactions gives a set of biochemical reactions precluded from the EMN. And the multi-input mode of *Avoid Reactions* is the same as *Sources* and *Avoid Compounds*.

Host Organism gives the genome-scale metabolic network model (GSMM) of the chassis microorganism that chosen for the production of the target product, the optional values are 'bsu', 'eco', 'kpn', 'llm', 'ppu', 'sce', 'syz' and 'N/A'. All these GSMMs were derived from BIGG [2], and the corresponding microorganisms of these GSMMs are described in Table 1. New GSMMs of some microorganisms (e.g., Corynebacterium glutamicum), if necessary, can be prepared and added to PyMiner by adding the new models' file in JSON format to path

‘../sourcedata/models/’ and appending the descriptions of these new models to ‘./datas/models.json’.

Table 1. All the GSMMs that integrated in PyMiner for pathway design.

Abbr.	Description
bsu	*Bacillus subtilis subsp. subtilis 168*
eco	*Escherichia coli K-12 MG1655*
kpn	*Klebsiella pneumoniae subsp. pneumoniae MGH 78578*
llm	*Lactococcus lactis subsp. cremoris MG1363*
ppu	*Pseudomonas putida KT2440*
sce	*Saccharomyces cerevisiae S288c*
syz	*Synechocystis sp. PCC 6803* (Cyanobacteria)
N/A	No chassis microorganism will be selected

Database gives the primary databases for pathway search, and the available databases include KEGG [3], MetaCyc [4], and KndPad (this study). The selected database will be distilled to construct an EMN on the first run. The original and distilled information for pathway search of these databases are saved in path ‘../sourcedata/’.

Maximum Length gives the maximum length (e.g., 4) of the potential pathways to restrict the number of retrieved pathways, while *Maximum Times(s)* gives the maximum time (e.g., 120s) to restrict the time that can be used for pathway search.

Similarity Difference Threshold gives the threshold ε (e.g., 0.1) used to generate main substrate-product pairs according the uniform similarity of all candidate substrate-product pairs. Furthermore, small value of ε expects a high atom utilization and a high atom conservation in single-step reaction.

Search Method gives the pathway search methods that applied, including 'bfs_naive' and 'dfs_naive', which respectively represent breadth-first search algorithm and depth-first search algorithm. And 'bfs_naive' method is recommended to be used firstly.

All pathways within *Maximum Length* will be retrieved if *Total* is checked, otherwise only pathways identical to *Maximum Length* will be identified. Similarly, only the shortest pathways of the target product will be accepted if *Shortest* is checked.

Retro Search gives users the freedom to choose the route search direction, that is searching from *Sources* to *Target* or searching from *Target* to *Sources*. Moreover, *Smart Search* provides a conditional search strategy (CSS) according to the local total out-degree (LTOD) of the start substrate and the local total in-degree (LTID) of the target product (described in Materials and methods section).

If *Infeasibility* is checked, biologically infeasible pathways (e.g., corresponding intermediate metabolites are absent from the selected microorganism) will not be dropped, and all pathways will entry the stage of evaluation.

The calculation of atom transfer route or main metabolic flux is a time-consuming process. Therefore, if *Atom Trace* and *Flux* are not checked, the potential synthetic pathways of the target product can be retrieved quickly. And if *Aerobic Culture* is not checked, the main metabolic flux will be performed under anaerobic conditions.

If materials (such as *Resource File*, *General Cofactors File* or *Atom Mapping Files*) used in PyMiner are changed, we can use *Update* checkbox to update the *Extracted metabolic network File*.

When all inputs are ready, *Start* push-button will be pressed to start the pathway design cycle.

3.2 **Pathways**

All candidate pathways of the target product (e.g., resveratrol) retrieved by PyMiner are demonstrated in *Pathways*, and the details of each pathway are listed in this table. If one pathway in this table is selected, more detailed information of the corresponding pathway will be demonstrated in table *Details*. In addition, for the convenience

of the communication with other researchers, the exported results are also provided in file './results/pathways/pathways.xlsx'.

Feasibility evaluates the biological feasibility of the corresponding pathway. If 'False' appears in this list cell, a more detailed explanation will be displayed in *I-Details* and *M-Details*.

TotalLength is the total length of the corresponding pathway.

EndoLength is the endogenous steps of the corresponding pathway.

HeterLength is the exogenous steps of the corresponding pathway.

InfLength is the biologically infeasible steps of the corresponding pathway.

AtomUtilization is the atom utilization of the initial substrate.

AtomConservation is the atom conservation of the target product.

MetabolicFlux is the main metabolic flux of the corresponding pathway, namely the maximum synthesis rate of the target product.

S-Details gives the string representation (composed of compounds and reactions) of the corresponding pathway.

3.3 **Tips**

Tips prints out all the necessary prompt information (S11 Fig) during the whole cycle of pathway design. During the parameter input phases, the input value to *Sources*, *Target, Avoid Compounds*, or *Avoid Reactions* will be displayed in *Tips* if Enter key is pressed. When start push-button is pressed, all the pre-set parameters are printed to Input portion in *Tips*. During or after the pathway search process, some useful information will be printed out to Search portion in *Tips*, such as the total number of the retrieved pathways. Moreover, *Clear* push-button can be used to clear the prompt box.

3.4 **Details**

As mentioned above, if one candidate pathway displayed in *Pathways* is selected, more detailed information will

be shown in *Details* (S10 Fig), including the potential transfer route (highlighted in green) of atoms from the start substrate to the target product, the compounds and the reactions with external links to their corresponding databases (e.g., KEGG, ChEBI [5], Rhea [6] and MetaCyc), and so on. Additionally, six types of reactions may be included in the graphical representation of the selected pathway. In details, green arrow indicates an endogenous irreversible or reversible reaction, blue arrow denotes an exogenous irreversible or reversible reaction, red arrow suggests a biologically infeasible irreversible or reversible reaction. What's more, if users are interested in the detailed atom transfer route of atoms from initial substrate to target product or the structure information of some compounds, mouse wheel could be used to zoom in/out the figures demonstrated in *Details*.

Step 4 Getting started with PyMiner

Here, several metabolic pathway design examples will be given to cast a glance on the application of PyMiner.

The exogenous pathways to resveratrol. If users are exclusively interested in the heterologous biosynthetic pathways of resveratrol in *E. coli*, then a null value can be provided to *Sources*. Compared to S10 Fig, MetaCyc instead of KEGG was adopted here (S2 Fig). In addition to the two reported pathways [7], that is, the second and the third pathways, a new pathway ranked first was identified which starts with 4-hydroxybenzoate. Additionally, other inputs employed in this example are illustrated in S2 Fig.

From D-xylose to xylitol. The example applied in this case study came from MRE [8]. As shown in S3 Fig, only one biosynthesis pathway from D-xylose to xylitol was extracted if *Total* in the *Inputs* was not checked. However, if *Total* is checked, three pathways within 2 steps will be retrieved [9]. In addition, other inputs utilized in this case study are demonstrated in S3 Fig.

From acetyl-CoA to Artemisinate. As an important precursor of antimalarial drug artemisinin, artemisinate has been used to semi-synthesize artemisinin [10, 11]. In this study, twelve pathways in total were identified by PyMiner, and the first one composed of eight endogenous steps and two exogenous steps (S4 Fig) has been

experimentally verified [10, 11]. This example indicates that if users have no prior knowledge on the length of the potential pathways, a lager value (e.g., 16) is suggested to be provided to *Maximum Length*, and to this end, *Shortest* should be checked.

From aldehydo-D-xylose to ethylene glycol. As shown in S9 Fig, when *Escherichia coli* (eco) was selected to synthesize ethylene glycol from aldehydo-D-xylose, two pathways were retrieved by PyMiner. The length of the second pathway (which was also retrieved by RouteSearch [12]) is 4. And aldehydo-D-xylose as the sole carbon which is missing in *Escherichia coli* should be added to the culture medium [13]. Furthermore, in order to retrieve these two pathways, *Infeasibility* was checked. And other inputs used in this case are shown in S9 Fig.

References

1. Ebrahim A, Lerman JA, Palsson BO, Hyduke DR. COBRApy: Constraints-based reconstruction and analysis for python. BMC systems biology. 2013; 7:74. doi: 10.1186/1752-0509-7-74 PMID: 23927696
2. King ZA, Lu J, Dräger A, Miller P, Federowicz S, Lerman JA, et al. BiGG Models: A platform for integrating, standardizing and sharing genome-scale models. Nucleic acids research. 2016; 44(D1):D515-D522. doi: 10.1093/nar/gkv1049 PMID: 26476456
3. Kanehisa M, Goto S, Sato Y, Kawashima M, Furumichi M, Tanabe M. Data, information, knowledge and principle: back to metabolism in KEGG. Nucleic acids research. 2014; 42(Database issue):D199-D205. doi: 10.1093/nar/gkt1076 PMID: 24214961
4. Caspi R, Billington R, Keseler IM, Kothari A, Krummenacker M, Midford PE, et al. The MetaCyc database of metabolic pathways and enzymes-a 2019 update. Nucleic acids research. 2020; 48(D1):D445-D453. doi: 10.1093/nar/gkz862 PMID: 31586394
5. Hastings J, Owen G, Dekker A, Ennis M, Kale N, Muthukrishnan V, et al. ChEBI in 2016: Improved services and an expanding collection of metabolites. Nucleic acids research. 2016; 44(D1):D1214-D1219. doi: 10.1093/nar/gkv1031 PMID: 26467479
6. Lombardot T, Morgat A, Axelsen KB, Aimo L, Hyka-Nouspikel N, Niknejad A, et al. Updates in Rhea: SPARQLing biochemical reaction data. Nucleic acids research. 2019; 47(D1):D596-D600. doi: 10.1093/nar/gky876 PMID: 30272209
7. Park JY, Lim JH, Ahn JH, Kim BG. Biosynthesis of resveratrol using metabolically engineered Escherichia coli. Applied biological chemistry. 2021; 64:20. doi: 10.116/s13765-021-00595-5
8. Kuwahara H, Alazmi M, Cui X, Gao X. MRE: a web tool to suggest foreign enzymes for the biosynthesis pathway design with competing endogenous reactions in mind. Nucleic acids research. 2016; 44(W1):W217-W225. doi: 10.1093/nar/gkw342 PMID: 27131375
9. Cirino PC, Chin JW, Ingram LO. Engineering Escherichia coli for xylitol production from glucose-xylose mixtures. Biotechnology and bioengineering. 2006; 95(6):1167-1176. doi: 10.1002/bit.21082 PMID: 16838379
10. Paddon CJ, Westfall PJ, Pitera DJ, Benjamin K, Fisher K, McPhee D, et al. High-level semi-synthetic production of the potent antimalarial artemisinin. Nature. 2013; 496(7446):528-532. doi: 10.1038/nature12051 PMID: 23575629
11. Paddon CJ, Keasling JD. Semi-synthetic artemisinin: a model for the use of synthetic biology in pharmaceutical development. Nature reviews microbiology. 2014; 12(5):355-367. doi: 10.1038/nrmicro3240 PMID: 24686413
12. Latendresse M, Krummenacker M, Karp PD. Optimal metabolic route search based on atom mappings. Bioinformatics. 2014; 30(14):2043-2050. doi: 10.1093/bioinformatics/btu150 PMID: 24642060
13. Liu H, Ramos KRM, Valdehuesa KNG, Nisola GM, Lee WK, Chung WJ. Biosynthesis of ethylene glycol in Escherichia coli. Applied microbiology and biotechnology. 2013; 97(8):3409-3417. doi: 10.1007/s00253-012-4618-7 PMID: 23233208
