# Supplementary material for: PyMiner: A method for metabolic pathway design based on the uniform similarity of substrate-product pairs and conditional search
Source: PLoS One. 2022 Apr 11;17(4):e0266783. doi: 10.1371/journal.pone.0266783 (PMC9000129; doi:10.1371/journal.pone.0266783)
Supplement: S2 File — (PDF) [file pone.0266783.s014.pdf]

Pseudo codes for
PyMiner: A Method for Metabolic Pathway Design Based on the Uniform Similarity of Substrate-Product Pairs and Conditional Search

Xinfang Song¶, Mingyu Dong¶ and Min Liu*

Department of Automation, Tsinghua University, Beijing 100084, China

* Corresponding author:

Email: lium@tsinghua.edu.cn (ML)

¶These authors contributed equally to this work.

1. Pseudo codes for "Constructing the extracted metabolic network (EMN)"

```
// 1) Remove compounds (COM) that belong to general cofactors (GC) from one reaction
// (RXN).
// 2) Calculate the uniform similarity (US) of all substrate-product pairs (SPP) according to
// the atom-atom mapping information (AAM) of RXN. Atoms that belong to GC and specific
// substructures (SS) are excluded.
// 3) Generate the main substrate-product pairs according the value of similarity difference
// threshold (ε).
GenerateRxnPair (RXN, GC, AMM, ε)
    // 1)
    For each COM in RXN Do
        If COM belongs to GC Then
            Remove COM from RXN.
            Record atoms belong to this COM by using AMM.
    // 2)
    For each substrate S in RXN Do
        Exclude atoms belong to GC and SS from S by using AMM.
        For each product P in RXN Do
            Exclude atoms belong to GC and SS from P by using AMM.
            Calculate and record the US between S and P.
    // 3)
    For each S in RXN Do
        max{US, ∀P}    // Pick and record the maximal value of US corresponding to S.
    For each P in RXN Do
        max{US, ∀S}    // Pick and record the maximal value of US corresponding to P.
    For each (S, P) pair in RXN Do
        If |max{US, ∀P} − US| ≤ ε And |max{US, ∀S} − US| ≤ ε Then
            Record this SPP.
    Return all SPP.
End GenerateRxnPair

// Generate the main substrate-product pairs of all reactions belong to the general metabolic
// network (GMN).
// All the main substrate-product pairs derived from GMN constitute the extracted metabolic
// network (EMN).
ConstructEMN (GMN, GC, AMM set, ε)
    For each RXN in GMN Do
        new SPP set ← GenerateRxnPair (RXN, GC, AMM, ε)
        Add new SPP set to EMN
    Return EMN
End ConstructEMN
```

2. Pseudo codes for "Conditional search strategy (CSS)"

```
// Conditional search strategy (CSS) based on the local total out degree (LTOD) of initial
// substrate (s) and the local total in degree (LTID) of target product (t).
// The search strategy (CSS) is applied only once to the initial substrate and the target
// product at the beginning of a search to help decide the search direction.
// Breadth-first search method (BFS) or depth-first search method is used.
// Biologically feasible and linear metabolic pathways need to meet two constraints.
ConSeaStr (s, t, EMN)
    Calculate the LTOD of initial substrate s by using EMN.
    Calculate the LTID of target product t by using EMN.
    If LTOD ≤ LTID Then
        Search from s to t within EMN by using BFS or DFS.
    Else
        Search from t to s within EMN by using BFS or DFS.
    Return all retrieved pathways.
End ConSeaStr
```

3. Pseudo codes for "Comprehensive evaluation method"

```
// The evaluation indexes include infeasible pathway length (l_inf), exogenous pathway length
// (l_exo), endogenous pathway length (l_endo), substrate-atom utilization (S_uti), product-atom
// conservation (T_con), and main metabolic flux (v_target) of target product.
// The format of one pathway is 's → RXN_1 → COM_1 → RXN_2 → COM_2 → ⋯ → t'.
CalIndex (pathway, AMM set, GSMM)
    // Initialization
    l_inf ← 0, l_exo ← 0, l_endo ← 0, S_uti ← 0, T_con ← 0, v_target ← NaN
    // The calculation of l_inf, l_exo and l_endo.
    For RXN in pathway Do
        If RXN belongs to GSMM Then
            l_endo ← l_endo + 1
        Else
            If all substrates of RXN belong to pathway or GSMM Then
                l_exo ← l_exo + 1
            Else
                l_inf ← l_inf + 1
    // The calculation of S_uti and T_con.
    For COM in pathway Do
        Calculate and record the substructure information of COM derived from s.
    Calculate the atoms of t derived from s.
    S_uti = N_A(s ∩ t)/N_A(s)
    T_con = N_A(s ∩ t)/N_A(t)
    // The calculation of v_target.
    If all substrates of all RXN belong to pathway or GSMM Then
        For COM in pathway Do
            Add pseudo metabolites to all reactions related to COM.
            Add pseudo reactions to GSMM corresponding to these pseudo metabolites.
        Calculate the main metabolic flux v_target by using COBRApy.
    //
    Return l_inf, l_exo, l_endo, S_uti, T_con and v_target.
End CalIndex


CompEvalMeth (pathway set, AMM set, GSMM)
    For pathway in pathway set Do
        pathway indexes ← CalIndex (pathway, AMM set, GSMM)
    Sort all retrieved pathways.
    Return the sorted pathways.
End CompEvalMeth
```
